# Synthesis of recovery patterns in microbial communities across environments

**DOI:** 10.1186/s40168-024-01802-3

**Published:** 2024-05-06

**Authors:** Stephanie D. Jurburg, Shane A. Blowes, Ashley Shade, Nico Eisenhauer, Jonathan M. Chase

**Affiliations:** 1 German Centre for Integrative Biodiversity Research (iDiv) Halle-Jena-Leipzig, 04103 Leipzig, Germany; 2 Department of Applied Microbial Ecology, Helmholtz Centre for Environmental Research - UFZ, Permoserstrasse 15, 04318 Leipzig, Germany; 3 Institute of Biology, Leipzig University, 04103 Leipzig, Germany; 4 Institute of Computer Science, Martin-Luther University Halle–Wittenberg, 06108 Halle (Saale), Halle Germany; 5 Laboratoire d’Ecologie Microbienne, UMR CNRS 5557, UMR INRAE 1418, VetAgro Sup, Universite Claude Bernard Lyon 1, 69622 Villeurbanne, France

**Keywords:** Community disturbance, Microbiome, Bacteria, Disturbance

## Abstract

**Background:**

Disturbances alter the diversity and composition of microbial communities. Yet a generalized empirical assessment of microbiome responses to disturbance across different environments is needed to understand the factors driving microbiome recovery, and the role of the environment in driving these patterns.

**Results:**

To this end, we combined null models with Bayesian generalized linear models to examine 86 time series of disturbed mammalian, aquatic, and soil microbiomes up to 50 days following disturbance. Overall, disturbances had the strongest effect on mammalian microbiomes, which lost taxa and later recovered their richness, but not their composition. In contrast, following disturbance, aquatic microbiomes tended away from their pre-disturbance composition over time. Surprisingly, across all environments, we found no evidence of increased compositional dispersion (i.e., variance) following disturbance, in contrast to the expectations of the Anna Karenina Principle.

**Conclusions:**

This is the first study to systematically compare secondary successional dynamics across disturbed microbiomes, using a consistent temporal scale and modeling approach. Our findings show that the recovery of microbiomes is environment-specific, and helps to reconcile existing, environment-specific research into a unified perspective.

Video Abstract

**Supplementary Information:**

The online version contains supplementary material available at 10.1186/s40168-024-01802-3.

## Background

Bacterial communities are ubiquitous [1], dynamic [2], and sensitive to environmental change [3, 4]. A wide range of literature explores microbiome responses to rapid environmental change in different environments [3], consistently revealing that microbial communities are affected by disturbance, and generally do not recover their pre-disturbance composition [5]. Historically, experimental procedures, designs, and hypotheses regarding the recovery of microbiomes following disturbance have developed in a largely field-specific manner (e.g., medical microbiology, soil microbiology, aquatic microbiology). Consequently, a comparison of community disturbance responses across microbial environments is lacking. Whether microbiomes from different environments exhibit responses to disturbance, and whether these responses are consistent with extant conceptual frameworks [6, 7] is a major gap in knowledge, especially considering growing anthropogenic pressures on microbial systems (e.g., pollutants, antibiotics, and climate extremes).

Properties of the microbial environment likely affect the dominant responses of microbiomes to disturbance, but empirical comparisons of recovery across environments are scarce [4]. Different microbial habitats have varying degrees of spatial and temporal heterogeneity, microbial species pool sizes, connectivity, and resource availability, all of which may affect community assembly processes [6], and likely result in different disturbance responses among environments. For example, animal gut microbiomes have relatively low diversity [1] and are dispersal-limited due to selective pressures associated with host physiology that likely influence the recovery of the resident microbial diversity. In contrast, soil microbiomes are extremely diverse, but poorly connected [8], likely affecting recolonization following disturbance. The lack of host-driven selection in these systems, combined with high diversity may result in communities composed of different taxon when compared to their pre-disturbance state.

**Fig. 1:**
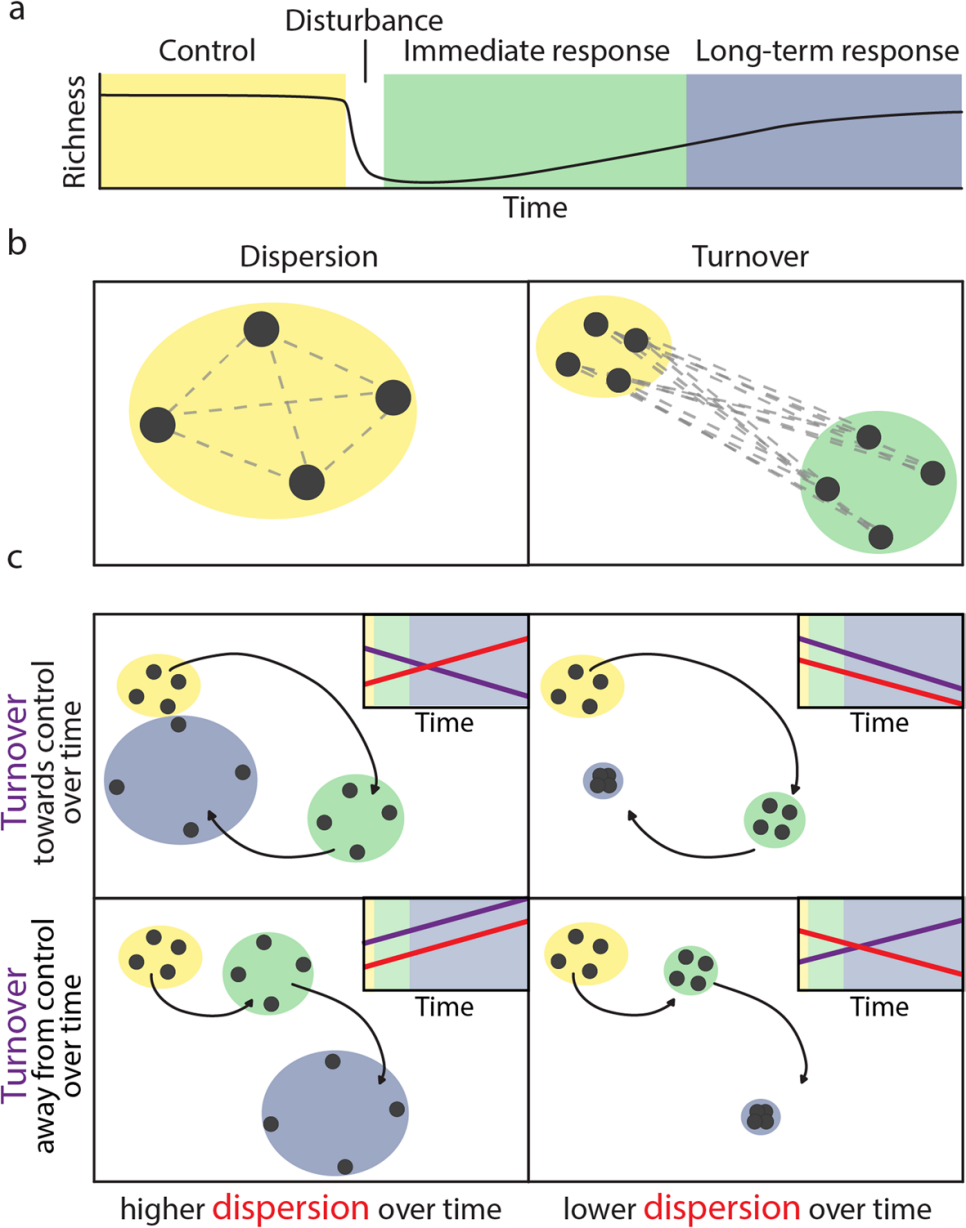
Microbial community dynamics after disturbance. The microbial community can be characterized in terms of its pre-disturbance state (yellow), its immediate response (green), and its long-term response (blue). Community richness can be monitored over time (**a**). In multivariate dissimilarity space (shown as ordinations in **b** and **c**, with samples as points), we can measure the dissimilarity between all experimental replicates in a study to quantify variability (**b** left, *dispersion*), and the dissimilarity between undisturbed communities and recovering communities to quantify overall changes in the community (**b** right, *turnover*). In **b**, gray dotted lines indicate pairwise comparisons included in each metric. Over time, disturbed community dispersion can increase (**c**, left) or decrease (**c**, right), and the community can tend towards the pre-disturbance state (negative turnover; **c**, top) or away from the pre-disturbance composition (positive turnover; **c**, bottom). For each set of samples, the centroid is indicated by an asterisk. In **a**, **b**, and **c**, color indicates stages of recovery. In **c**, insets indicate how turnover (purple) and dispersion (red) are visualized as change over time

Assessments of microbiome recovery often rely on indicator measurements that are environment-specific (e.g., host health in host-associated microbiomes or plant productivity in soil microbiomes), hindering the comparison of microbial disturbance responses across environments. By considering changes in diversity at multiple spatial scales (i.e., within and among samples) and the role of spatial connectivity in these responses, the metacommunity framework [9] can help to synthesize and explicitly compare microbial community responses to disturbance across environments, and in turn provide new insights into the role of the environment in shaping these responses [4]. To this end, publicly available 16S rRNA gene amplicon sequences can be leveraged to assess bacterial community responses as changes in bacterial richness (the number of taxa present in a sample) and composition (variation in taxon relative abundance between samples). Generally, we expect that across environments, community richness will decrease (Fig. 1a), as has been found across both aquatic and terrestrial ecosystems [10] We also expect that community composition will change immediately after the disturbance, due for example to differential mortality and an altered competitive landscape [5]. However, environmental change does not consistently result in decreased richness [11]. Additionally, in microbes, disturbances may involve the addition of novel taxa (e.g., with sewage sludge amendments to soil [12]), which may result in richness increases. Over longer time scales following disturbance, richness may either fail to fully recover (at least within the period observed; e.g., [13]), recover fully [14], or even be higher following disturbance [15].

Community composition is often a more robust indicator of biodiversity change than richness [11]. Compositional changes can be assessed in terms of compositional variation among local communities [16], or *dispersion*, and the extent to which the community recovers to its pre-disturbance composition, or *turnover* (Fig. 1b). Following disturbance, dispersion can decrease, for example, if a stressor is selective and leaves only tolerant taxa to persist. Alternatively, dispersion can increase, for example, if the stressor is non-selective, or more generally if taxa that persist following disturbance differ [17]. In microbiomes, the Anna Karenina Principle (AKP), derived primarily from the observation of host-associated communities, posits that healthy microbiomes are more stable, and thus less variable than disturbed ones [18].

Given enough time, we expect the same taxa that dominated prior to a disturbance to recover their original abundances [4], especially in host-associated microbiomes, which can be modulated by the host [19]. However, under some circumstances (e.g., strong or long disturbances, or invasion by novel taxa [20, 21]), it is also possible that the disturbance could permanently alter relative abundance patterns in the community [22, 23], resulting in communities that tend away from their pre-disturbance composition over time. Across environments, microbiomes have been shown to recover towards (negative turnover, e.g., [14, 24]), or to drift away from (positive turnover, e.g., [25]), their pre-disturbance compositions. Importantly, both changes in dispersion and turnover can arise from changes in richness alone and null models have been developed that allow for the measurement of compositional change independent of changes in community richness [26].

Meta-analyses focusing on the undisturbed temporal dynamics of microbial communities have shown consistent patterns across systems [2, 5, 27], but temporal disturbance responses have received less attention [4]. To this end, we performed a synthetic analysis of the time series of disturbed aquatic, mammal-associated, and soil microbiomes. Across environments, we compared the initial response and subsequent recovery from disturbance in terms of community richness, dispersion, and turnover, and used null models to disentangle whether the observed changes in dispersion and turnover were due to changes in richness. Given the rapid rates of compositional turnover in microbiomes [28], we focused on 29 studies that repeatedly sampled the microbiomes within 50 days post-disturbance.

## Methods

### Dataset selection

Using Google Scholar and Web of Science search engines (a list of keywords is available as Supplementary Materials), we collated bacterial studies from systems where an experimental disturbance was imposed, and 16S rRNA gene amplicon sequencing datasets were available. Specifically, we chose studies that (1) were sequenced in Illumina or IonTorrent platforms; (2) sequenced the V3–V4 regions of the 16S rRNA gene; (3) were published after 2014; (4) repeatedly sampled microbial communities following a discrete disturbance or environmental change; (5) included samples from before the disturbance (i.e., controls), at least one (replicated) sample within a week after disturbance, and at least one (replicated) sample within a month after disturbance; and, (6) included experimental triplicates (i.e., three samples per time point). Criteria 1–3 ensured that the sequencing techniques were comparable between studies, and reduced the biases associated with sampling different regions of the 16S rRNA gene [29]. Importantly, downstream analyses adopted a synthetic framework (i.e., we reprocessed sequences using a single approach described below), and samples from different studies were not combined. We applied criteria 4–6 to examine variation in rates of compositional change across environments. Criterion 6 ensured that the variability of the microbiomes at each time point could be measured. We defined a disturbance causally, as a “discrete, rapid environmental change” [30]. We excluded datasets for which raw sequencing data were not publicly available and stopped data collection in October 2020. In all, datasets from 29 studies matched our criteria [14, 23, 31–54], see Table S1 for all datasets). We grouped these time series into three environmental categories: aquatic, mammal-associated, and soil microbiomes (including rhizosphere microbiomes). To further explore the role of disturbance type on the observed phenomena, we categorized disturbances according to their effect on the community as previously done in macroecology [16]. Categories included mortality-inducing treatments (e.g., heat, azoxystrobin, ciprofloxacin, mechanical removal), mortality-inducing treatments combined with a microbial invasion (e.g., cefuroxime and *Clostridium difficile*), mortality-inducing treatments combined with nutrient additions (e.g., heat and fertilizer additions), drought, invasions (e.g., the addition of *Pseudomonas* or *C. difficile*), metal pollution (e.g., cadmium additions), nutrient additions (nitrate, chitin, diesel), nutrient additions including potential invasions (e.g., the addition of wastewater, the addition of diesel and a bacterial consortium), and PAH contamination.

### Sequence reprocessing and functional inference

Raw 16S rRNA gene amplicon data and metadata were obtained from the NCBI Sequence Read Archives with the exception of two datasets, one of which came from another database, and the other was obtained directly from the authors (see Table S1 for accession numbers). We reprocessed sequences in R 3.4.3 [55] using the *dada2* package [56], and a conservative approach. To account for the different sequence qualities across datasets and to improve comparability in the reprocessed data, each dataset was inspected and reprocessed separately, and downstream statistical analyses accounted for between-study differences. Prior to processing, we visually inspected two samples per study with the *plotQualityProfile* to determine whether the reads had been merged prior to archiving, and to confirm that primers were not present. We only used forward reads because reverse reads were not available for all studies. Following inspection, we trimmed and truncated sequences on a study-by-study basis (see Table S1 for trimming and truncation lengths) to preserve a 90-bp segment, the minimum recommended in the Earth Microbiome Project protocols [1] (and the maximum possible for studies that used Illumina HiSeq machines). We acknowledge that 90 bp is shorter than the length that is often used in amplicon sequencing studies and that longer segments would have detected higher microbial diversity; however, our aim was to compare diversity patterns across studies, for which short read lengths are suitable [57]. Similar to downstream rarefaction, trimming all segments to the same length ensured a comparable degree of biodiversity detection across studies [57].

We filtered, dereplicated, and chimera‐checked each read using standard workflow parameters [58]. While we did not use taxonomic assignments in our analyses or compare amplicon sequence variants (ASVs, 100% sequence identity) across datasets, we assigned reads to ASVs with the SILVA v.132 training set [59] to remove non-bacterial ASVs. Unassigned, bacterial ASVs (i.e., those classified as Bacteria) were preserved. Details about the percentage of reads lost at each step of sequence processing, per study, are included in Fig. S1. As the samples included in these studies had a wide range of sequencing depths across samples (independent of the study environment), we randomly subsampled each sample to 1500 reads per sample to obtain a similar degree of biodiversity detection across studies. To ensure that our findings were not affected by observation depth, we additionally ran all analyses in parallel using the deepest possible observation depth (with a lower bound of 1500 reads per sample) for each study (Table S1). As our findings were consistent regardless of standardization (Fig. S2), we present only the results from the global rarefaction (i.e., 1500 reads per sample for all samples). To examine the completeness of each sample relative to the total richness in a community, we calculated sample completeness [60] using the *BetaC* package [61]. On average, our samples represented 0.96 ± 0.05 (mean ± sd) of the community. We removed any time points that had fewer than three experimental replicates for each time series. We coded time series so that time (days) ≥ 0 occurred after disturbance, and time < 0 denoted the pre-disturbance community.

### Calculation of richness and turnover metrics

To examine variation in diversity across environments we calculated metrics that quantify diversity within samples (richness), and variation in taxon composition between samples (turnover). We calculated richness and turnover metrics using the *phyloseq* package’s data structure [62]*.* We calculated species richness as the number of unique ASVs per sample (Hill *q* = 0), and Inverse Simpson’s index (Hill *q* = 2 [63]). We used Bray–Curtis dissimilarity to quantify two aspects of compositional variation. First, to describe the compositional variation between samples collected at the same time point, we calculated dispersion as the pairwise Bray–Curtis dissimilarity between all combinations of experimental replicates for each time point within each time series. For studies that resampled the same experimental unit (e.g., host organism or microcosm) over time, we excluded pairwise comparisons between samples from the same experimental units. Second, to quantify how composition changed following disturbance, we calculated turnover using pairwise dissimilarities between all control samples (i.e., pre-disturbance) and all subsequent replicate samples at each time point following disturbance. Using this approach, communities that recover their pre-disturbance state will have a negative slope estimate through time, while communities that become increasingly different from the pre-disturbance community over time will have a positive slope estimate (Fig. 1).

Because compositional changes can be due to changes in richness alone, we used a null model to disentangle compositional changes from changes in richness. We randomly permuted abundance values within each sample 1000 times, preserving the number of taxa (i.e., richness) for each sample, and recalculated turnover and dispersion metrics for each matrix to derive a null expectation for each. For both metrics, *Z*-scores were calculated as $\frac{{{\text{u}}}^{{\text{observed}}}-{\mu }^{{\text{expected}}}}{{\sigma }^{{\text{expected}}}}$, where ${\mu }^{{\text{expected}}}$ is the mean of the resamples, and ${\sigma }^{{\text{expected}}}$ is the standard deviation. Z-scores are a powerful method to explore dissimilarities as deviations from a null expectation [64], perform particularly well for long-tailed microbiome data, and are recommended over subtraction-based dissimilarity partitioning methods [65]. Statistical analyses evaluated dissimilarity and *Z*-score values in parallel. Significant (95% credible interval) patterns observed in both dissimilarity and *Z*-score data were attributed to changes in community richness, while significant patterns observed only in the *Z*-score data were attributed to changes in the relative abundance of taxa within the community. We present models fit to the raw dissimilarity metrics (i.e., Bray–Curtis) in the main text, and report where they differed from analyses of the *Z*-scores, which are presented in full in Figs. S6 and S9. All code for bioinformatics processing and null models is available at https://github.com/drcarrot/DisturbanceSynthesis.

### Statistical analyses

We fit generalized linear models to assess how richness, dispersion, and turnover change in response to disturbances using Bayesian methods and the *brms* package [62], and detailed information about each model is provided in the “Supplementary methods” section. We performed all analyses at the ASV level. To quantify the immediate response of richness and dispersion to disturbance, we used before-after analyses that compared data from prior to the disturbance to samples taken < 4 days post-disturbance; to determine whether responses differed between environments (i.e., aquatic, mammal, soil), we included an interaction between the before-after and environment categorical covariates. Five studies were excluded from the before-after analyses due to a lack of samples (Table S1). To quantify how richness and dispersion changed through time following disturbance, we fit models to data from the first 50 days post-disturbance only (i.e., pre-disturbance samples were not included). Finally, to examine how composition changed from pre- to post-disturbance, we fit models to turnover that quantified compositional changes between the pre-disturbance controls and samples taken in the first 50 days post-disturbance. To determine whether changes following disturbance differed between environments, all-time series models included an interaction between time and environment. Time (in days) was fit as a continuous covariate and was centered by subtracting the mean duration from all observations prior to modeling. We fit all models with the same, hierarchical grouping (or random-effects) structure: to account for methodological variation between studies, we included varying intercepts for each study in all models; and, because many studies included more than one disturbance type (e.g., [35]), we included varying slopes and intercepts for time series within studies (i.e., one time series per disturbance type). Models fit species richness (i.e., the before-after and time series models) assumed a negative-binomial error distribution and a log-link function. In addition to the parameters and the grouping structure described above, the shape parameter of the negative-binomial distribution (that estimates aggregation) was also allowed to vary among studies. Models fit raw values of dispersion and turnover assumed Beta error, a logit-link function, and the precision parameter was allowed to vary among studies. Models fit to Z-transformed dispersion and turnover assumed Gaussian error, an identity link, and to account for heteroskedasticity residual variation (i.e., the sigma parameter) was modeled as a function of the environment and allowed to vary among studies. The modeled responses and means per group, as well as the 95% CI, are depicted together with the data where applicable. For each comparison and for each environment, we identified time series that exhibited an upward or downward trend if the 97.5% CI did not overlap with zero, and neutral otherwise.

For Bayesian inference and estimates of uncertainty, we fit models using the Hamiltonian Monte Carlo (HMC) sampler Stan [66], which was coded using the *brms* package [67]. We used weakly regularizing priors, and visual inspection of the HMC chains showed excellent convergence. All code for statistical analyses is available at https://github.com/sablowes/microbiome-disturbance.

## Results

**Fig. 2:**
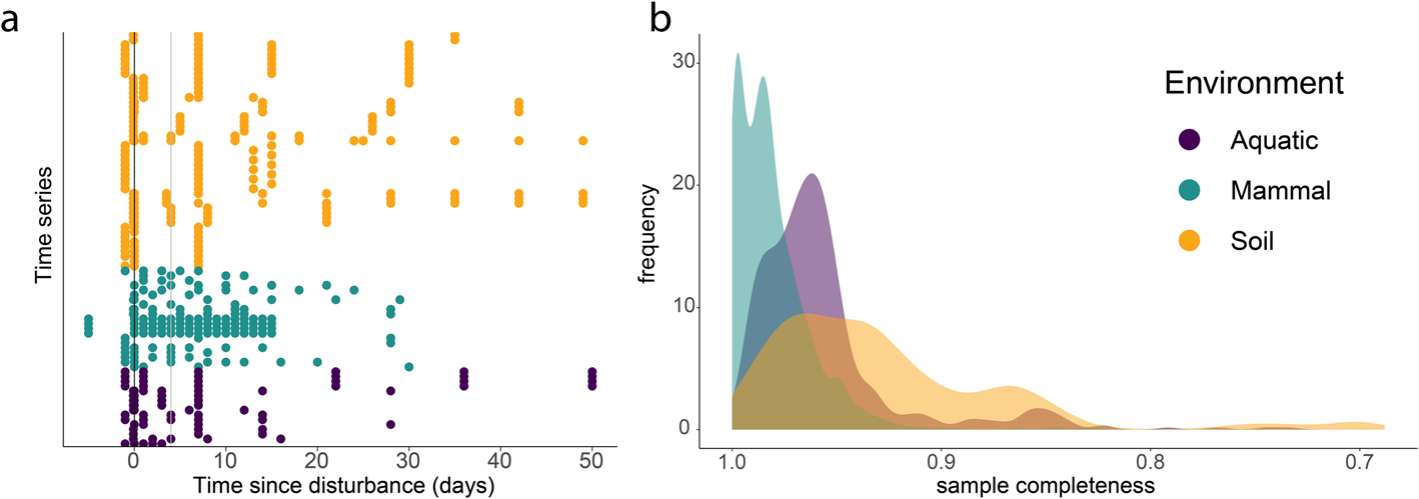
Samples used in this meta-analysis. We selected a time series which had control samples and multiple samples after disturbance (**a**). A vertical black line denotes a disturbance event in all cases; samples taken on the day of the disturbance (before or after) are shown along this line. A vertical gray line indicates the fourth day after the disturbance. Studies which had not sampled the recovering microbiome within < 4 days after disturbance were excluded from assessments of the immediate impacts of disturbance on richness and dispersion. All samples were standardized to 1500 observations per sample, and had an average sample completeness > 90% (**b**). In **b**, the sample completeness for all samples included in the synthesis is shown as a histogram. Sample completeness, or the proportion of the community that belongs to sampled taxa [60], was estimated according to [61]

Our final dataset included 2588 samples in 86-time series from 29 studies (Table S1) belonging to soil micro- and mesocosms (*n* = 49), seawater mesocosms (*n* = 16), and mammalian microbiomes (*n* = 21) that were sampled multiple times within 50 days after disturbance (Fig. 2a). Across all samples, we detected 56,480 ASVs. Sample completeness was highest in mammalian microbiomes (0.98 ± 0.02; mean ± sd), lowest and most variable in soil microbiomes (0.93 ± 0.06), and was significantly different between environments (ANOVA, *F* = 475.1, *p* < 0.001, Fig. 2b).

### Richness in disturbed and recovering microbiomes

**Fig. 3:**
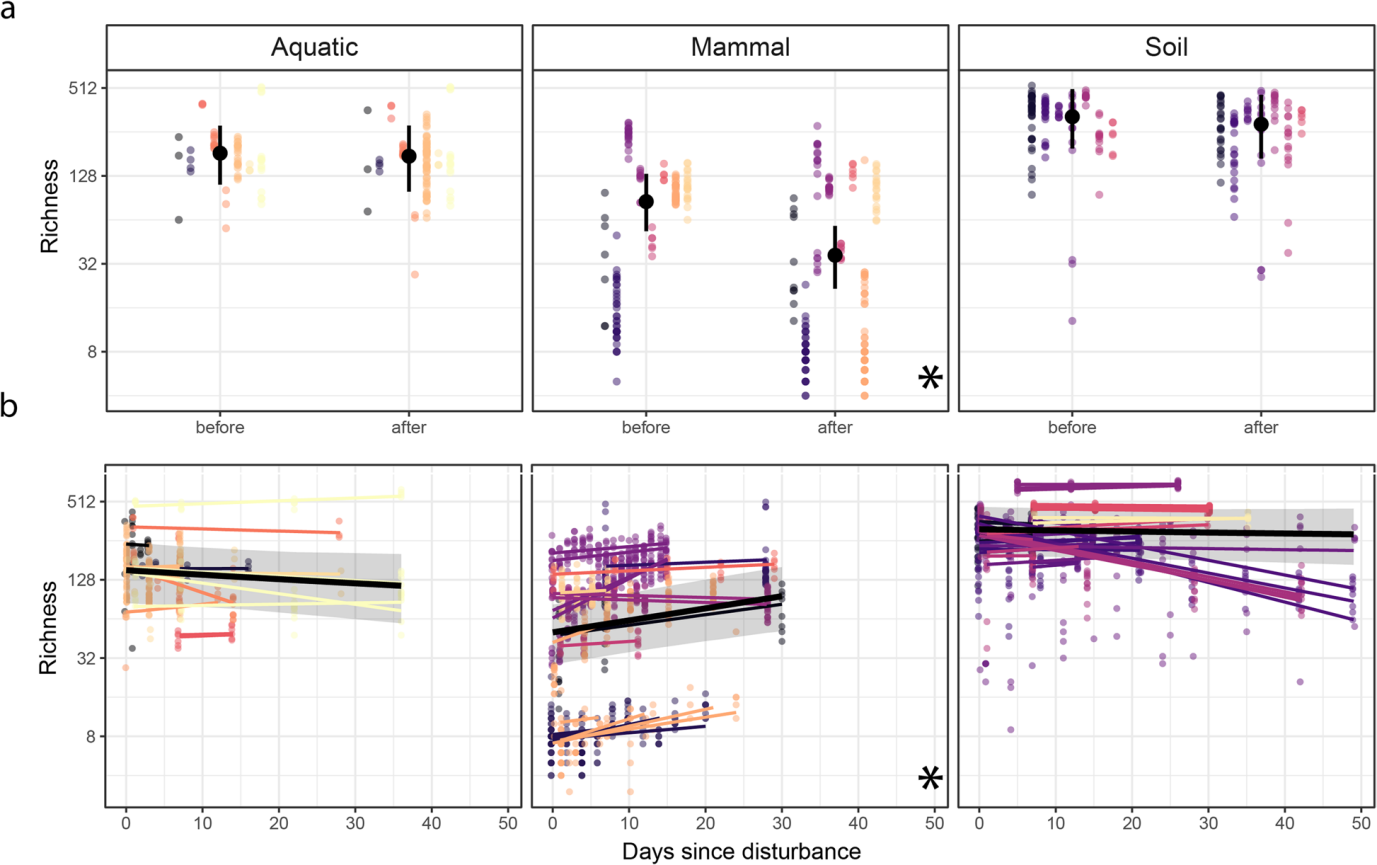
The effect of disturbance on microbiome richness, immediately (< 4 days) after disturbance (**a**), and over 50 days of recovery (**b**). Richness was calculated as the number of observed taxa in each sample and is presented in a log_2_-transformed *y*-axis. Points represent samples and are colored by study. In **a**, solid black points indicate the modeled mean across time series per environment with a 95% CI indicated by error bars. In **b**, thin regression lines for each time series are colored by study, and the solid black line shows the modeled mean response across time series per environment. The 95% CI is displayed as a gray-shaded area, and environments for which overall trends deviate from zero are indicated with an asterisk (*) on the bottom right corner

Prior to disturbance, mean richness was highest in soil microbiomes with 327 ASVs [95% CI 196–506], followed by aquatic 184 [111–281], and mammalian 86 [51–133] microbiomes (Fig. 3a). While all environments exhibited decreases in microbiome richness following disturbance, only the decrease in the mammalian microbiomes statistically differed from zero, and all mammalian time series (*n* = 19 time series) exhibited a downward richness trend (Table 1). This pattern was primarily driven by time series which employed disturbances that likely caused mortality, or those that introduced an invasion, or a combination of both (Fig. S3). In contrast, all aquatic time series (*n* = 14) and most soil time series (*n* = 20) with the exception of four exhibited neutral trends (Table 1).

**Table 1:** Microbiome disturbance responses per environment

	Aquatic			Mammal			Soil		
	↓	−	↑	↓	−	↑	↓	−	↑
Immediate richness change	0	14	0	19	0	0	4	20	0
Temporal richness change	5	11	0	0	1	19	6	41	0
Immediate dispersion change	0	10	4	2	13	2	0	29	4
Temporal dispersion change	0	11	5	13	7	0	15	31	1
Turnover	0	0	16	14	6	0	2	29	16

For each comparison and for each environment, we identified time series that exhibited an upward or downward trend if the 97.5% CI did not overlap with zero, and neutral otherwise. Numbers indicate the numbers of time series responding according to a specific parameter (e.g., immediate richness change)

On average, the post-disturbance richness in mammalian microbiomes was approximately 43% of that found pre-disturbance (Fig. 3a), and over time, richness increased consistently at a rate of approximately 2% (1–3%) per day (Fig. 3b), a phenomenon that was observed across disturbance types and was present in all mammal time series (*n* = 19) except for one that exhibited neutral trends. In general, the mammalian microbiomes that lost the most richness after disturbance also recovered this richness most rapidly over the following 50 days (Fig. S4). In contrast, no overall patterns were observed in the richness in aquatic and soil time series, although they exhibited either neutral responses or (*n* = 11 and *n* = 41 for aquatic and soil time series) or the continued loss of richness over time (*n* = 5 and *n* = 6, respectively, Table S1). These results were consistent when alpha diversity recovery was assessed as inverse Simpson’s index (Fig. S5).

### Dispersion and turnover

**Fig. 4:**
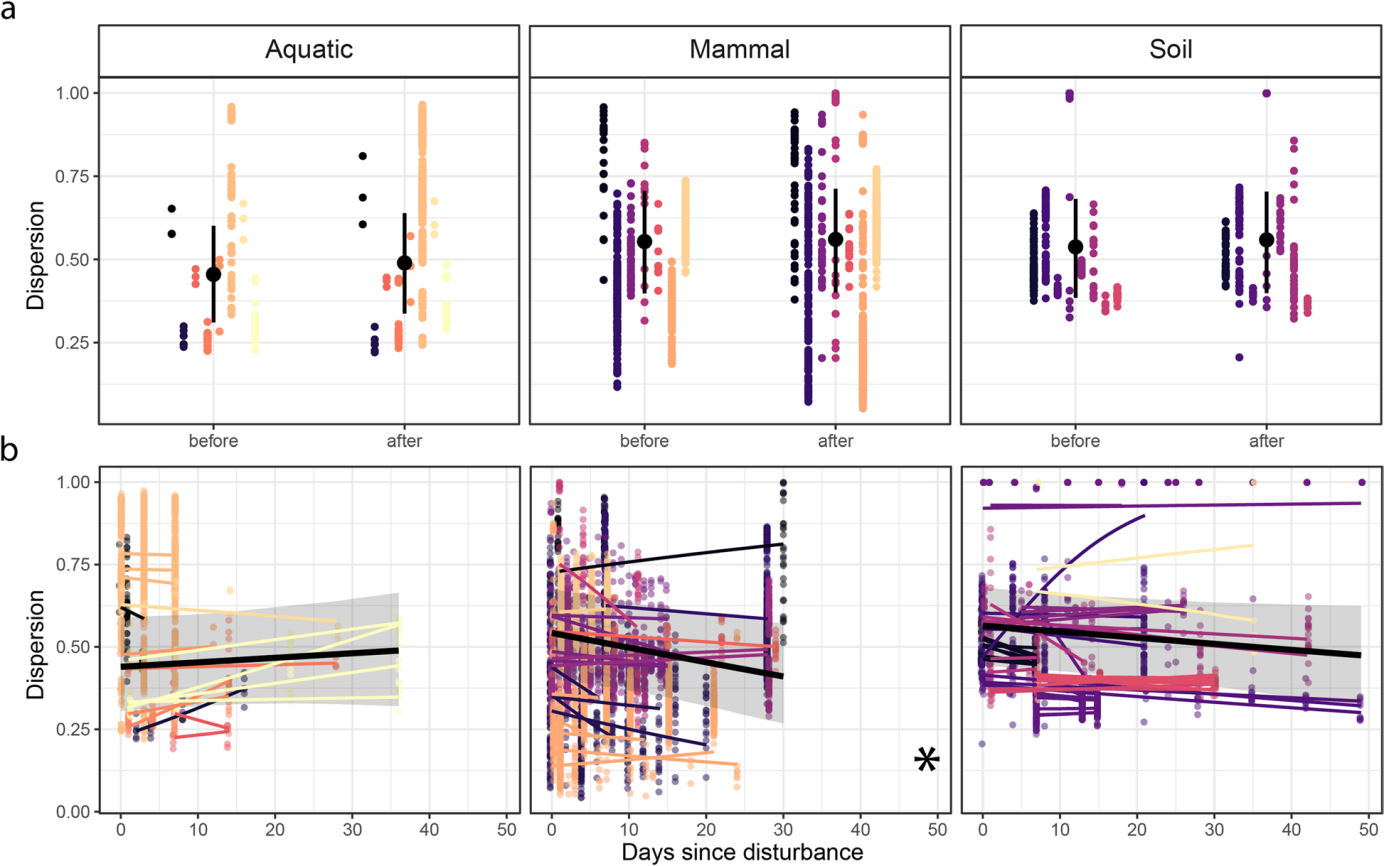
The effect of disturbance on microbiome dispersion, immediately (< 4 days) after disturbance (**a**), and over 50 days of recovery (**b**). Dispersion was calculated as the pairwise Bray–Curtis dissimilarity between replicates for each time point within each time series, and each point is a pairwise comparison, colored by study. In **a**, solid black points indicate the modeled mean across time series per environment with a 95% CI indicated by error bars. In **b**, thin regression lines for each time series are colored by study, and the solid black line shows the modeled mean response across time series per environment. The 95% CI of the overall response in each environment is displayed as a gray-shaded area, and environments for which overall trends deviate from zero are indicated with an asterisk (*) on the bottom right corner

All microbial communities were under dispersed relative to the null expectation, and 97% of *Z*-scores were negative. All of the lowest *Z*-score values (< − 400) belonged to mouse microbiomes, for which we detected fewer than 30 ASVs. On average, dispersion did not change immediately after disturbance for any environment (Fig. 4a, Table S2). However, we found a decrease through time following the disturbance in dispersion values for mammalian microbiomes (Fig. 4b), though this pattern was not present in the *Z*-scores (Fig. S6), indicating reduced compositional variation was associated with a reduction in richness, rather than changes in relative abundances. The strongest responses were from microbiomes exposed to invasion (*n* = 1), mortality (*n* = 10), or a mixture of both (*n* = 8, Fig. S7). Most mammal time series (*n* = 13) exhibited a decreasing dispersion over time, while 7 exhibited neutral dynamics (Table 1). Similarly, soil time series exhibited mostly decreasing (*n* = 15) or neutral (*n* = 31) dispersion dynamics, with only one-time series increasing in dispersion over time. In contrast, aquatic time series exhibited either neutral (*n* = 11) or increasing (*n* = 5) dispersion over time.

**Fig. 5:**
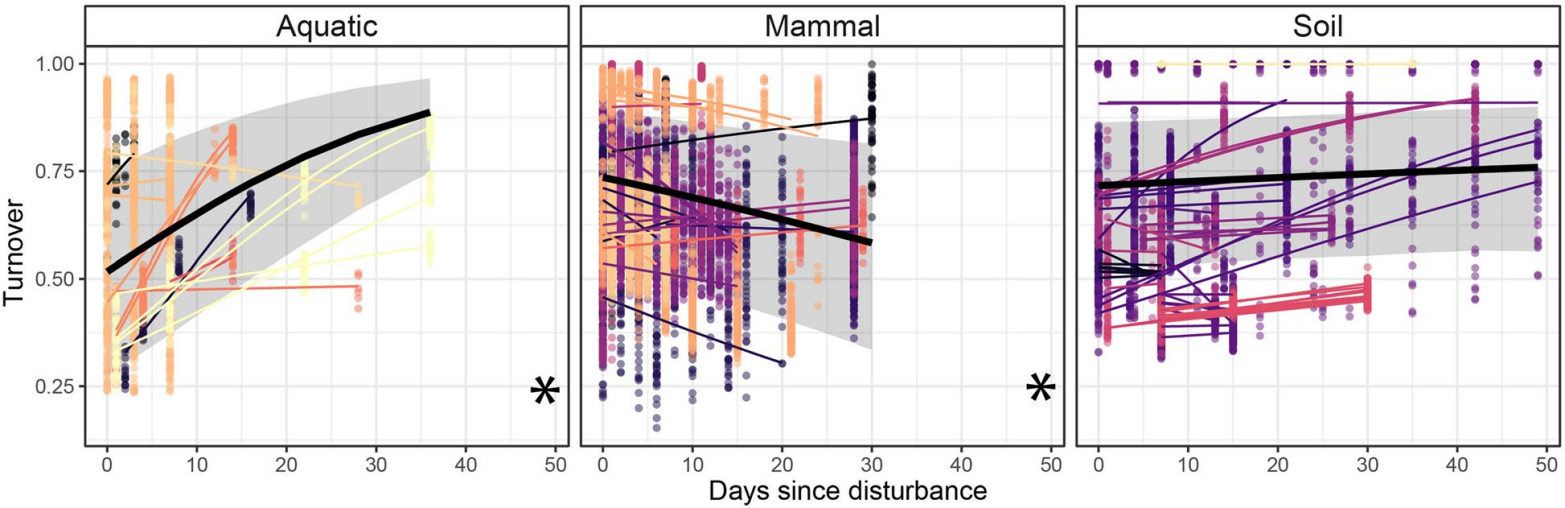
The effect of disturbance on community recovery is environment-dependent. For each time series, recovery was calculated as the pairwise dissimilarity between post-disturbance samples and pre-disturbance controls. Each point is a pairwise comparison, colored by study. Microbiomes which recover their pre-disturbance state will exhibit negative slopes; microbiomes which continue to drift away from their pre-disturbance composition over time will exhibit positive slopes. Thin regression lines for each time series are colored by study, and a solid black line indicates the modeled mean response across time series per environment. The 95% CI is displayed as a gray shaded area, and environments for which overall trends deviate from zero are indicated with an asterisk (*) on the bottom right corner

We found environment-specific turnover between composition pre- and post-disturbance. On average, mammalian microbiomes exhibited negative turnover, and most time series (*n* = 14) tended to recover toward their pre-disturbance composition (Fig. 5, Table 1). This pattern was consistent across disturbance types and was strongest for microbiomes subjected to invasion (*n* = 1), mortality (*n* = 10), or a combination of both (*n* = 8, Fig. S8). Importantly, negative turnover was not found when assessed with *Z*-scores (Fig. S9), indicating that recovery occurred through an increase in richness, not due to the recovery of relative abundances. In contrast, following disturbance, aquatic microbiomes exhibited positive turnover, tending away from their pre-disturbance controls over time. This pattern was present in all-time series (*n* = 16), and was consistent whether raw values (Fig. 5) or *Z*-scores were modeled (Fig. S8), indicating that changes in the identity and relative abundance of taxa, rather than simply changes in the number of taxa in the system were responsible for this drift away from a pre-disturbance composition. While all-time series followed this response regardless of the type of disturbance, PAH and metal-contaminated microbiomes (*n* = 1 for each) exhibited the strongest response (Fig. S8). Notably, while no consistent responses were found in soil, most time series exhibited positive (*n* = 16) or neutral (*n* = 29) turnover, with only two-time series tending towards recovery (i.e., negative turnover).

**Fig. 6:**
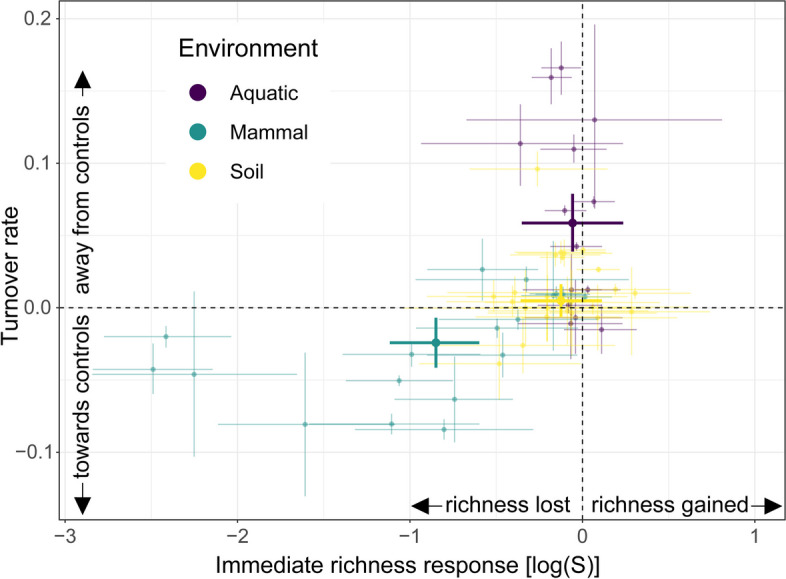
Relationships between the immediate effect of a disturbance on richness and a microbiome’s long-term recovery of composition vary among environments. Each point is a time series, colored by its environment. Immediate richness responses were calculated as the before-after effect of disturbance on log-transformed community richness (Fig. 3a). Turnover rates were calculated as the modeled slope estimates of logit-transformed turnover over time. Error bars show the 95% CI for both metrics. Large points indicate the mean responses per environment

Finally, to examine the relationship between the immediate disturbance responses (i.e., the strength of the disturbance) and compositional changes over time subsequent to the disturbance, we plotted rates of temporal turnover as a function of the magnitude of the immediate (< 4 days after disturbance) changes in richness (Fig. 6). This relationship was environment-dependent. Aquatic microbiomes predominantly exhibited no immediate richness responses to disturbance and positive turnover thereafter (i.e., composition moved away from pre-disturbance controls); mammalian microbiomes exhibited an immediate loss of richness and a negative turnover (i.e., recovery toward pre-disturbance composition); and soil microbiomes exhibited very weak or no responses in terms of both immediate richness responses and turnover following the disturbance (Fig. 6). This pattern was consistent, but weaker when turnover *Z*-scores were modeled, especially for mammalian microbiomes (Fig. S10).

## Discussion

We synthesized metabarcoding data to show how microbial community responses to disturbance vary across three environments at time scales that are relevant to microbiome turnover rates and bacterial life histories [28, 68]. We focused on the richness, dispersion, and turnover of microbiomes recovering from 86 different disturbances in three different environments, and further partitioned the latter two into shifts caused by changes in richness or in the relative distribution of taxa in order to shed light on the ecological processes driving microbial recovery. We found environment-specific responses: aquatic microbiomes tended away from their pre-disturbance composition following disturbance, while mammalian microbiomes tended to recover towards their pre-disturbance state. Soil microbiomes exhibited no clear patterns. Furthermore, we found no indication that disturbances increased dispersion in any environment, in contrast with the Anna Karenina Principle (AKP), and instead found the opposite pattern, especially in mammalian microbiomes. These findings highlight consistent response patterns within environments and consistent differences between environments.

Contrary to our expectation, we only found modest losses in richness following disturbance. On average, only mammalian microbiomes experienced statistically significant richness loss. This loss likely underscores the efficacy of antibiotics, which were used in 76% of mammalian microbiome time series, often in combination with an invader such as *C. difficile* [23, 35] Disturbances in soil and aquatic environments in our study were dominated by nutrient additions (e.g., inorganic nitrogen and phosphorus inputs in aquatic microbiomes, [49] or humic acid amendments in soil, [45]), which are not directly expected to decrease richness. Surprisingly, we did not record any instance of a nutrient addition increasing richness in these systems, but this may be because all the experimental systems selected in the meta-analysis were partially closed to dispersal from the local environment (e.g., microcosms and mesocosm).

Despite their strong initial response to disturbance, mammalian microbiomes exhibited a clear and rapid trend toward recovery over time. Our null model analyses showed that richness changes were largely responsible for the decreases in community dispersion (i.e., more similar taxa composition) and negative turnover following the disturbance, suggesting that in mammals, disturbance generally resulted in the loss of specific taxa followed by a rapid recolonization by these taxa. Given the absence of this pattern in soil or aquatic microbiota, our findings suggest role of the host in modulating and perhaps accelerating the recovery of the resident microbiota. Host behaviors such as eating [69] and socializing [70] may function as mechanisms of active dispersal, and together with the immune system may act as a selective pressure [19], resulting in recovered microbiomes that resemble the undisturbed communities. Several studies have demonstrated the high variability in host responses to disturbance [71] and the dependence of these responses on the environment [72]; however, by comparing these responses with those found in other environments, we found that host-associated microbiomes exhibited the strongest and most consistent responses to disturbance.

Surprisingly, aquatic microbiomes tended to become more dissimilar from their pre-disturbance compositions over time. This pattern may be due to the high connectivity and constant mixing of resources (i.e., nutrients) in aquatic microbiomes [73]. Due to the different experimental designs included in this synthesis, it was not possible to determine whether the communities were generally drifting towards a specific composition (i.e., an alternative stable state [74]).

In contrast, in the highly heterogeneous soil environment, microbiomes did not exhibit strong responses to disturbance. Nevertheless, similarities with the other environments were present: in all environments, we recorded no instances of soil microbiomes increasing in richness immediately following disturbance. Like in aquatic microbiomes, we also found no instances of soil microbiomes recovering their richness over time following disturbance, or of dispersion decreasing immediately after disturbance. We also found that a substantial portion of the soil time series tended away from their pre-disturbance state. As in mammalian microbiomes, we found several instances of microbiome turnover tending towards decreased dispersion over time.

In the above cases, most time series in soil exhibited neutral responses (i.e., no detectable trend), however. This pattern could be due to the extreme diversity and heterogeneity found in this system [75], or due to technical limitations of this study. Nevertheless, standardizing the data to the maximum depth for each time series yielded identical results, suggesting that higher resolution may be necessary to capture community recovery in soils and disentangle the role of rare taxa from stochasticity. The conservative approaches we employed for the selection, processing, and analysis of the data aimed to facilitate cross-study comparisons, but limited the contribution of rare taxa (i.e., those with low relative abundance) in our analyses of diversity change. Recognizing these limitations, we focused on the dominant taxa, using abundance-weighted metrics (Bray–Curtis). This likely impacted our analysis of soil most strongly, as soil microbiomes had the highest overall richness and lowest sample completeness estimates, and rare taxa are important sources of variation in soil microbiomes [76, 77].

It is likely that our sample size (*n* = 86 time series) and statistical methods (applied to standardize and enable direct comparison across habitats) have together provided a broader analysis than was previously achieved from habitat-specific studies. We found no indication that dispersion increases immediately or over time following disturbance, in any environment, in direct contrast with the AKP. The AKP proposes that dysbiotic microbiomes exhibit an increased host-to-host variation [18]. Importantly, our synthesis did not include measures of dysbiosis, as these were not consistently available and the definition of dysbiosis can vary widely. Instead, we compared the microbiomes to their pre-disturbance state and found that disturbance does not consistently increase dispersion, at least in the dominant portion of the community. While changes in dispersion are often reported in the microbial literature [78–80], dispersion is generally measured as pairwise Bray–Curtis dissimilarity among experimental or field replicates, and confounds changes in richness with compositional changes [26, 81]. We found that, in general, when dispersion decreased (i.e., in mammals), it was due to decreasing species richness in the community, not due to changes in the relative abundance of community members. We also found that in the absence of a host, soil and aquatic microbiomes tended to shift away from their pre-disturbance conformation, suggesting that environmental microbiomes are less prone to recovery than mammalian ones. Taken together, this synthesis sheds light on similarities across environments and highlights the role of the host in microbiome recovery.

## Conclusion

Our work highlights the need to reconsider the definition of disturbance in the microbiome [82]. We included a wide range of disturbances, and categorized them according to a framework that considered the direct effect of the disturbance on the microbial community and that largely echoes similar categorizations in macroecology (e.g., [10, 16]). For example, when sterilized, organic amendments represent a novel source of resources, but when applied unsterilized, they also potentially include an invasive community, a scenario that deviates from the classic invasion literature [83]. Furthermore, selective disturbances (e.g., antibiotics) remove similar taxa across experimental replicates, resulting in the homogenization of microbiomes, and decreasing dispersion [47]. In contrast, disturbances that affect taxa randomly could lead to the microbiomes becoming more dissimilar, increasing the influence of ecological drift, and consequently, compositional dispersion. The duration of disturbances also varied, especially relative to bacterial life histories and ecologies [28]. Pulse disturbances which last multiple days may encompass multiple life cycles for many microbial taxa. Similarly, disturbances which may be considered long-term changes for macro-organisms (i.e., oil pollution), may represent short-term resource pulses for oil-degrading bacteria. In a world in which microbiomes are exposed to increasing disturbance pressures, developing a set of descriptors for disturbances based on their effect on the microbiome’s niche space and competitive landscape is urgently needed.

Our study reconciles several hypotheses that have been proposed for microbiomes, with different hypotheses supported in different environments. First, we find strong support for the tendency to drift away from the pre-disturbance state in aquatic systems, and mild support in soil systems [74]. Second, we find a strong tendency towards recovery in mammalian microbiomes, characterized by the loss of specific taxa during disturbance and their return thereafter. Third, we find little general evidence for changes in compositional dispersion (after accounting for changes in richness) following disturbance, in contrast to the AKP. Our work focused on community-level responses to disturbances across microbiomes, but did not delve into the responses of specific taxa due to the differences in sequencing techniques (and especially primer choice among studies [84]. Future work may focus on smaller subsets of data that use consistent techniques to identify responsive taxa. Our results highlight how richness alone does not capture complex microbiome dynamics, similar to findings in broader ecology [11]. Further work is needed to distinguish the consequences of selective versus non-selective disturbances (e.g., those that impact certain populations versus those that indiscriminately impact all populations) on microbiome responses. Overall, this work provides a new empirical perspective on the dynamics and generalities of microbiome disturbance responses that are supported by directly comparable metrics, equivalent temporal scales among datasets, and a consistent modeling approach. It suggests that with comparisons of standardized diversity measures, responses that were previously believed to be applicable to all microbiomes (i.e., the AKP) are not present and that the environment (especially the host) is a key determinant of the microbiome of both the response to, and recovery from, disturbance.

### Supplementary Information

**Additional file 1: Supplementary methods**. Literature Search and model descriptions. **Table S1.** Accession numbers and links to all sequences reused in this work and their processing parameters. **Table S2.** Slope estimates for models comparing immediate changes in dispersion following disturbance, calculated on Bray–Curtis values and null model outputs. **Figure S1.** Proportion of reads preserved after quality filtering (a), chimera checking (b), and selection of bacterial reads (c). **Figure S2.** Models fit to data standardized across studies and to data standardized within studies yield very similar parameter estimates. Each panel shows the fixed effect estimates for models fit to (a) richness before-after disturbance, (b) richness change through time following disturbance, (c) dispersion before-after disturbance, (d) dispersion (z-score) before-after disturbance, (e) dispersion change through time following disturbance, (f) dispersion (z-score) change through time following disturbance, (g) turnover change through time following disturbance, and (h) turnover (z-score) change through time following disturbance. **Figure S3.** Posterior distributions of the immediate response in richness to disturbance, separated by disturbance type and microbial realm. **Figure S4.** The immediate effect of a disturbance on richness was only related to the rate of recovery of richness in mammals. **Figure S5.** Slope and interval estimate of richness (Hill q_0_, purple) and inverse Simpson’s index (Hill q_2_, blue) immediately following disturbance (a) and over time (b). **Figure S6.** The effect of disturbance on microbiome dispersion, immediately (< 4 days) after disturbance (a), and over 50 days of recovery (b). **Figure S7.** Posterior distribution of temporal response of dispersion to disturbance, separated by disturbance type and microbial realm. **Figure S8.** Posterior distribution of temporal response of turnover to disturbance, separated by disturbance type and microbial realm. **Figure S9.** The effect of disturbance on turnover. **Figure S10.** Relationships between the immediate effect of a disturbance on richness and a microbiome’s long-term recovery of composition vary among environments.

## Data Availability

No datasets were generated or analysed during the current study.
