# Supplementary material for: Synthesis of recovery patterns in microbial communities across environments
Source: Microbiome. 2024 May 6;12:79. doi: 10.1186/s40168-024-01802-3 (PMC11071242; doi:10.1186/s40168-024-01802-3)
Supplement: Supplementary file 2 — Additional file 1: Supplementary methods. Literature Search and model descriptions. Table S1. Accession numbers and links to all sequences reused in this work and their processing parameters. Table S2. Slope estimates for models comparing immediate changes in dispersion following disturbance, calculated on Bray–Curtis values and null model outputs. Figure S1. Proportion of reads preserved after quality filtering (a), chimera checking (b), and selection of bacterial reads (c). Figure S2. Models fit to data standardized across studies and to data standardized within studies yield very similar parameter estimates. Each panel shows the fixed effect estimates for models fit to (a) richness before-after disturbance, (b) richness change through time following disturbance, (c) dispersion before-after disturbance, (d) dispersion (z-score) before-after disturbance, (e) dispersion change through time following disturbance, (f) dispersion (z-score) change through time following disturbance, (g) turnover change through time following disturbance, and (h) turnover (z-score) change through time following disturbance. Figure S3. Posterior distributions of the immediate response in richness to disturbance, separated by disturbance type and microbial realm. Figure S4. The immediate effect of a disturbance on richness was only related to the rate of recovery of richness in mammals. Figure S5. Slope and interval estimate of richness (Hill q0, purple) and inverse Simpson’s index (Hill q2, blue) immediately following disturbance (a) and over time (b). Figure S6. The effect of disturbance on microbiome dispersion, immediately (< 4 days) after disturbance (a), and over 50 days of recovery (b). Figure S7. Posterior distribution of temporal response of dispersion to disturbance, separated by disturbance type and microbial realm. Figure S8. Posterior distribution of temporal response of turnover to disturbance, separated by disturbance type and microbial realm. Figure S9. The effect of distur [file 40168_2024_1802_MOESM1_ESM.zip › Supplementary Materials.docx]

**Supplementary Information**

**Supplementary Methods: Literature Search**

The following keyword searches were performed in October-November 2018. For Web of Science, the syntax was *(TS=((microb* OR bacteri*) AND (temporal OR succession OR time OR dynamics) AND V4 AND miseq)) AND LANGUAGE:(English) AND DOCUMENT TYPES:(Article); Timespan: Last 5 years. Indexes: SCI-EXPANDED, SSCI, A&HCI, CPCI-S, CPCI-SSH, BKCI-S, BKCI-SSH, ESCI, CCR-EXPANDED, IC.* For Google Scholar, the search syntax was *"V4", Miseq, accession, temporal, OR time, OR succession, OR disturbance, OR stress, OR dynamics -v3*. Results were filtered to include only publications since 2015. Search results were initially checked for temporal sampling intervals, the region of the 16S rRNA gene sequenced and the availability of raw sequence data prior to further inspection.

**Supplementary Methods: Statistical models**

The model to evaluate the immediate response to disturbance for richness in time series *j* nested in study *i* assumed negative binomial error and a log-link function, and took the form:

$$S_{ij}\sim nbinom\left( \mu_{ij},\phi_{i} \right)$$

$$\log\left( u_{ij} \right)=\beta_{0}+\beta_{0i}+\beta_{0ij}+\beta_{1}{env}_{ij}+\beta_{2}after+{(\beta}_{3}+ \beta_{3ij})after*{env}_{ij}$$

$$\log\left( \phi_{i} \right)=\beta_{0}^{\phi}+\beta_{0i}^{\phi}$$

$$\beta_{0i}\sim N(0,\sigma_{i})$$

$$\beta_{0i}^{\phi}\sim N(0,\sigma_{i}^{\phi})$$

$${[\beta}_{0ij},\beta_{3ij}]'\sim MVN(0,\boldsymbol{SRS)}$$

$$\boldsymbol{S}=\left[ \begin{matrix} \sigma_{0ij} & 0 \\ 0 & \sigma_{3ij} \end{matrix} \right],$$

$\boldsymbol{R}=\left[ \begin{matrix} 1 & \rho_{\sigma_{0ij}\sigma_{3ij}} \\ \rho_{\sigma_{0ij}\sigma_{3ij}} & 1 \end{matrix} \right]$,

where $\beta_{0}$ is the intercept and estimates the average richness in aquatic environment; $\beta_{0i}$ is a varying study-level intercept with zero mean and $\sigma_{i}$ standard deviation; ${env}_{i}$ are indicator variables for the mammal and soil environments, and $\beta_{1}$ estimates the departures from the intercept (aquatic environment) for mammal and soil environments; *after* is an indicator for whether an observation is after the disturbance has been applied, and $\beta_{2}$ estimates the average richness in aquatic environments within four days of the disturbance; $\beta_{3}$ estimates the departures from the aquatic average after the disturbance for the mammal and soil environments; $\beta_{0ij}$and $\beta_{3ij}$ are varying intercepts and slopes for time series (j) nested with studies (i) and were drawn from multivariate normal (MVN) distribution, that allowed for correlations (***R*** matrix) between the standard deviations $\sigma_{0ij}$ and $\sigma_{3ij}$ (***S*** matrix); $\phi_{i}$

is a study-level shape parameter, with overall average $\beta_{0}^{\phi}$and varying-study level intercepts $\beta_{0i}^{\phi}$ with zero mean and $\sigma_{i}^{\phi}$standard deviation.

The model to evaluate the immediate response to disturbance for dispersion (*disp*) in time series *j* nested in study *i* assumed beta error and a logit-link function, and took the form:

$${disp}_{ij}\sim beta\left( \mu_{ij},\phi_{i} \right)$$

$$logit \left( u_{ij} \right)=\beta_{0}+\beta_{0i}+\beta_{0ij}+\beta_{1}{env}_{ij}+\beta_{2}after+{(\beta}_{3}+ \beta_{3ij})after*{env}_{ij}$$

$$\log\left( \phi_{i} \right)=\beta_{0}^{\phi}+\beta_{0i}^{\phi}$$

$$\beta_{0i}\sim N(0,\sigma_{i})$$

$$\beta_{0i}^{\phi}\sim N(0,\sigma_{i}^{\phi})$$

$${[\beta}_{0ij},\beta_{3ij}]'\sim MVN(0,\boldsymbol{SRS)}$$

$$\boldsymbol{S}=\left[ \begin{matrix} \sigma_{0ij} & 0 \\ 0 & \sigma_{3ij} \end{matrix} \right],$$

$\boldsymbol{R}=\left[ \begin{matrix} 1 & \rho_{\sigma_{0ij}\sigma_{3ij}} \\ \rho_{\sigma_{0ij}\sigma_{3ij}} & 1 \end{matrix} \right]$,

where $\beta_{0}$ is the intercept and estimates the average dispersion in aquatic environment; $\beta_{0i}$ is a varying study-level intercept with zero mean and $\sigma_{i}$ standard deviation; ${env}_{i}$ are indicator variables for the mammal and soil environments, and $\beta_{1}$ estimates the departures from the intercept (aquatic environment) for mammal and soil environments; *after* is an indicator for whether an observation is after the disturbance has been applied, and $\beta_{2}$ estimates the average dispersion in aquatic environments within four days of the disturbance; $\beta_{3}$ estimates the departures from the aquatic average after the disturbance for the mammal and soil environments; $\beta_{0ij}$and $\beta_{3ij}$ are varying intercepts and slopes for time series (j) nested with studies (i) and were drawn from multivariate normal (MVN) distribution, that allowed for correlations (***R*** matrix) between the standard deviations $\sigma_{0ij}$ and $\sigma_{3ij}$ (***S*** matrix); $\phi_{i}$is a study-level precision parameter, with overall average $\beta_{0}^{\phi}$and varying-study level intercepts $\beta_{0i}^{\phi}$ with zero mean and $\sigma_{i}^{\phi}$standard deviation.

To estimate how richness in time series j nested with study I recovered following disturbance we fit a model that assumed negative binomial error and a log-link function:

$$S_{ij}\sim nbinom\left( \mu_{ij},\phi_{i} \right)$$

$$\log\left( u_{ij} \right)=\beta_{0}+\beta_{0i}+\beta_{0ij}+\beta_{1}{env}_{ij}+\beta_{2}time+{(\beta}_{3}+ \beta_{3ij})time*{env}_{ij}$$

$$\log\left( \phi_{i} \right)=\beta_{0}^{\phi}+\beta_{0i}^{\phi}$$

$$\beta_{0i}\sim N(0,\sigma_{i})$$

$$\beta_{0i}^{\phi}\sim N(0,\sigma_{i}^{\phi})$$

$${[\beta}_{0ij},\beta_{3ij}]'\sim MVN(0,\boldsymbol{SRS)}$$

$$\boldsymbol{S}=\left[ \begin{matrix} \sigma_{0ij} & 0 \\ 0 & \sigma_{3ij} \end{matrix} \right],$$

$\boldsymbol{R}=\left[ \begin{matrix} 1 & \rho_{\sigma_{0ij}\sigma_{3ij}} \\ \rho_{\sigma_{0ij}\sigma_{3ij}} & 1 \end{matrix} \right]$,

where $\beta_{0}$ is the intercept and estimates the average richness in aquatic environment; $\beta_{0i}$ is a varying study-level intercept with zero mean and $\sigma_{i}$ standard deviation; ${env}_{i}$ are indicator variables for the mammal and soil environments, and $\beta_{1}$ estimates the departures from the intercept (aquatic environment) for mammal and soil environments; *time* codes the time since disturbance in days, which we centered by subtracting the mean from each observation before fitting model (and back-transformed when plotting results), and $\beta_{2}$ estimates the average rate of change of richness in aquatic environments following disturbance; $\beta_{3}$ estimates the departures from the aquatic average rate of change for the mammal and soil environments; $\beta_{0ij}$and $\beta_{3ij}$ are varying intercepts and slopes for time series (j) nested with studies (i) and were drawn from multivariate normal (MVN) distribution, that allowed for correlations (***R*** matrix) between the standard deviations $\sigma_{0ij}$ and $\sigma_{3ij}$ (***S*** matrix); $\phi_{i}$ is a study-level shape parameter, with overall average $\beta_{0}^{\phi}$and varying-study level intercepts $\beta_{0i}^{\phi}$ with zero mean and $\sigma_{i}^{\phi}$standard deviation.

To estimate how dispersion in time series *j* nested with study *i* recovered following disturbance we fit a model that assumed beta error and a logit-link function:

$${disp}_{ij}\sim beta\left( \mu_{ij},\phi_{i} \right)$$

$$logit \left( u_{ij} \right)=\beta_{0}+\beta_{0i}+\beta_{0ij}+\beta_{1}{env}_{ij}+\beta_{2}time+{(\beta}_{3}+ \beta_{3ij})time*{env}_{ij}$$

$$\log\left( \phi_{i} \right)=\beta_{0}^{\phi}+\beta_{0i}^{\phi}$$

$$\beta_{0i}\sim N(0,\sigma_{i})$$

$$\beta_{0i}^{\phi}\sim N(0,\sigma_{i}^{\phi})$$

$${[\beta}_{0ij},\beta_{3ij}]'\sim MVN(0,\boldsymbol{SRS)}$$

$$\boldsymbol{S}=\left[ \begin{matrix} \sigma_{0ij} & 0 \\ 0 & \sigma_{3ij} \end{matrix} \right],$$

$\boldsymbol{R}=\left[ \begin{matrix} 1 & \rho_{\sigma_{0ij}\sigma_{3ij}} \\ \rho_{\sigma_{0ij}\sigma_{3ij}} & 1 \end{matrix} \right]$,

where $\beta_{0}$ is the intercept and estimates the average dispersion in aquatic environments; $\beta_{0i}$ is a varying study-level intercept with zero mean and $\sigma_{i}$ standard deviation; ${env}_{i}$ are indicator variables for the mammal and soil environments, and $\beta_{1}$ estimates the departures from the intercept (aquatic environment) for average dispersion in mammal and soil environments; *time* codes the time since disturbance in days, which we centered by subtracting the mean from each observation before fitting model (and back-transformed when plotting results), and $\beta_{2}$ estimates the average rate of change in dispersion for aquatic environments following disturbance; $\beta_{3}$ estimates the departures from the aquatic average rate of change for the mammal and soil environments; $\beta_{0ij}$and $\beta_{3ij}$ are varying intercepts and slopes for time series (j) nested with studies (i) and were drawn from multivariate normal (MVN) distribution, that allowed for correlations (***R*** matrix) between the standard deviations $\sigma_{0ij}$ and $\sigma_{3ij}$ (***S*** matrix); $\phi_{i}$ is a study-level precision parameter, with overall average $\beta_{0}^{\phi}$and varying-study level intercepts $\beta_{0i}^{\phi}$ with zero mean and $\sigma_{i}^{\phi}$standard deviation.

Finally, to estimate how composition changed from pre-to-post disturbance, we fit models to turnover (*turn*) in time series *j* from study *i* that assumed beta error and a logit-link function:

$${turn}_{ij}\sim beta\left( \mu_{ij},\phi_{i} \right)$$

$$logit \left( u_{ij} \right)=\beta_{0}+\beta_{0i}+\beta_{0ij}+\beta_{1}{env}_{ij}+\beta_{2}time+{(\beta}_{3}+ \beta_{3ij})time*{env}_{ij}$$

$$\log\left( \phi_{i} \right)=\beta_{0}^{\phi}+\beta_{0i}^{\phi}$$

$$\beta_{0i}\sim N(0,\sigma_{i})$$

$$\beta_{0i}^{\phi}\sim N(0,\sigma_{i}^{\phi})$$

$${[\beta}_{0ij},\beta_{3ij}]'\sim MVN(0,\boldsymbol{SRS)}$$

$$\boldsymbol{S}=\left[ \begin{matrix} \sigma_{0ij} & 0 \\ 0 & \sigma_{3ij} \end{matrix} \right],$$

$\boldsymbol{R}=\left[ \begin{matrix} 1 & \rho_{\sigma_{0ij}\sigma_{3ij}} \\ \rho_{\sigma_{0ij}\sigma_{3ij}} & 1 \end{matrix} \right]$,

where $\beta_{0}$ is the intercept and estimates the average turnover in aquatic environments; $\beta_{0i}$ is a varying study-level intercept with zero mean and $\sigma_{i}$ standard deviation; ${env}_{i}$ are indicator variables for the mammal and soil environments, and $\beta_{1}$ estimates the departures from the intercept (aquatic environment) for average turnover in mammal and soil environments; *time* codes the time since disturbance in days, which we centered by subtracting the mean from each observation before fitting model (and back-transformed when plotting results), and $\beta_{2}$ estimates the average rate of change in turnover for aquatic environments following disturbance; $\beta_{3}$ estimates the departures from the aquatic average rate of change for the mammal and soil environments; $\beta_{0ij}$and $\beta_{3ij}$ are varying intercepts and slopes for time series (j) nested with studies (i) and were drawn from multivariate normal (MVN) distribution, that allowed for correlations (***R*** matrix) between the standard deviations $\sigma_{0ij}$ and $\sigma_{3ij}$ (***S*** matrix); $\phi_{i}$ is a study-level precision parameter, with overall average $\beta_{0}^{\phi}$and varying-study level intercepts $\beta_{0i}^{\phi}$ with zero mean and $\sigma_{i}^{\phi}$standard deviation.

The models fit to Z-transformed dispersion and turnover had the similar structure to those detailed above, only we replaced the beta error distributions with Gaussian error distributions, used identity link functions, and added environment specific intercepts to the predictors for residual variation, along with the varying study-level intercepts.

[Table 1 is presented separately as an excel file.](https://docs.google.com/spreadsheets/d/1o5Uvzh5Q4VE7HXhpeMt-hs5wfkybDwRZ/edit?usp=sharing&ouid=113332841508967711520&rtpof=true&sd=true)

Table S2. Slope estimates for models comparing immediate changes in dispersion following disturbance, calculated on Bray-Curtis values and null model outputs.

|  | **Slope** | **Estimate** | **Q2.5** | **Q97.5** |
| --- | --- | --- | --- | --- |
| Bray- curtis values | Aquatic | 0.638 | -0.978 | 2.242 |
|  | Mammalian | 0.783 | -2.709 | 4.246 |
|  | Soil | 0.772 | -2.519 | 4.081 |
| null model outputs | Aquatic | 0.141 | -0.042 | 0.319 |
|  | Mammalian | 0.027 | -0.399 | 0.451 |
|  | Soil | 0.087 | -0.322 | 0.502 |


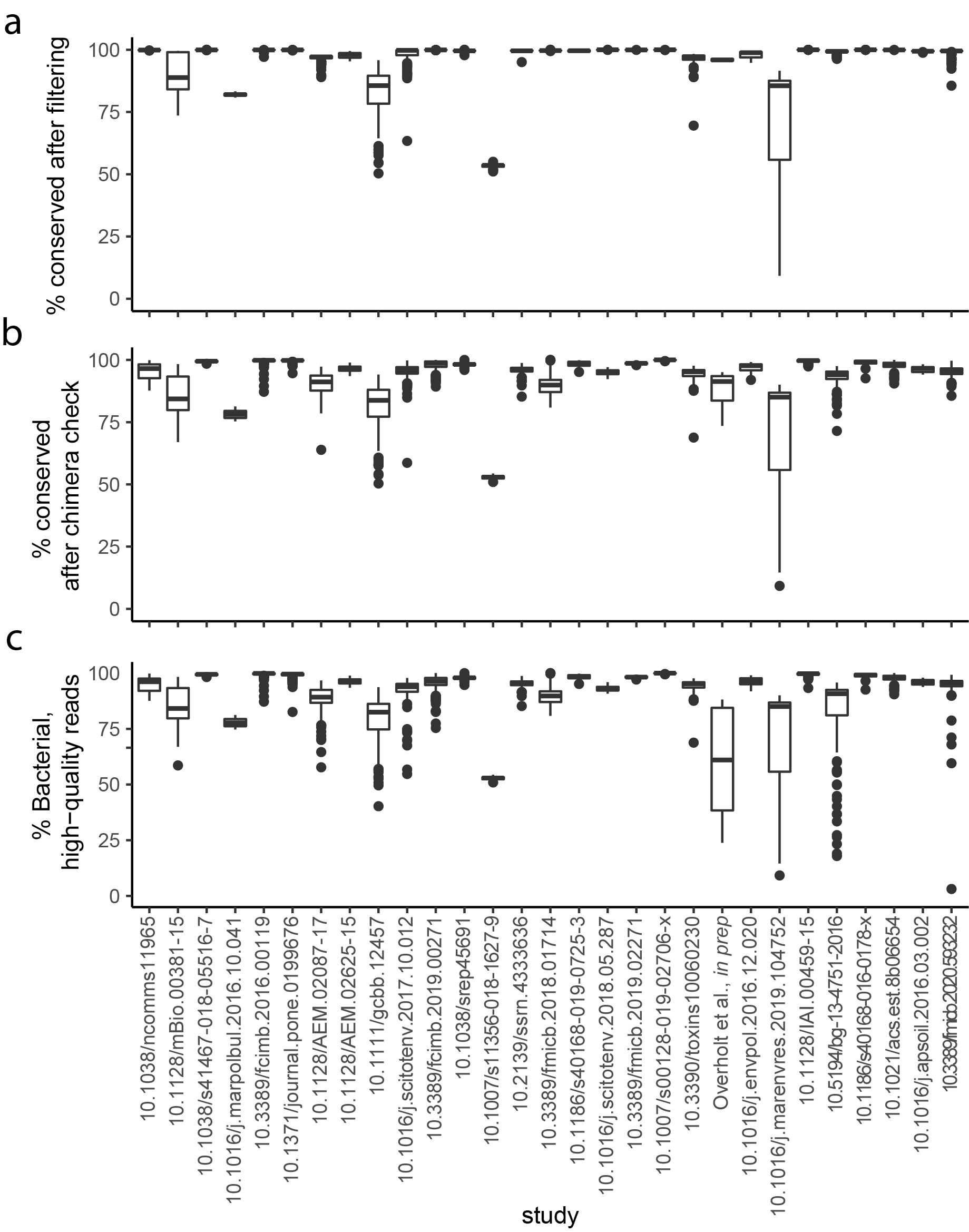


Figure S1. Proportion of reads preserved after quality filtering (a), chimera checking (b), and selection of bacterial reads (c). All data is presented as a proportion of the number of reads originally recovered from public databases, and presented per study. Studies are labeled with their corresponding DOIs, and additional per-study information is found in Table S1.


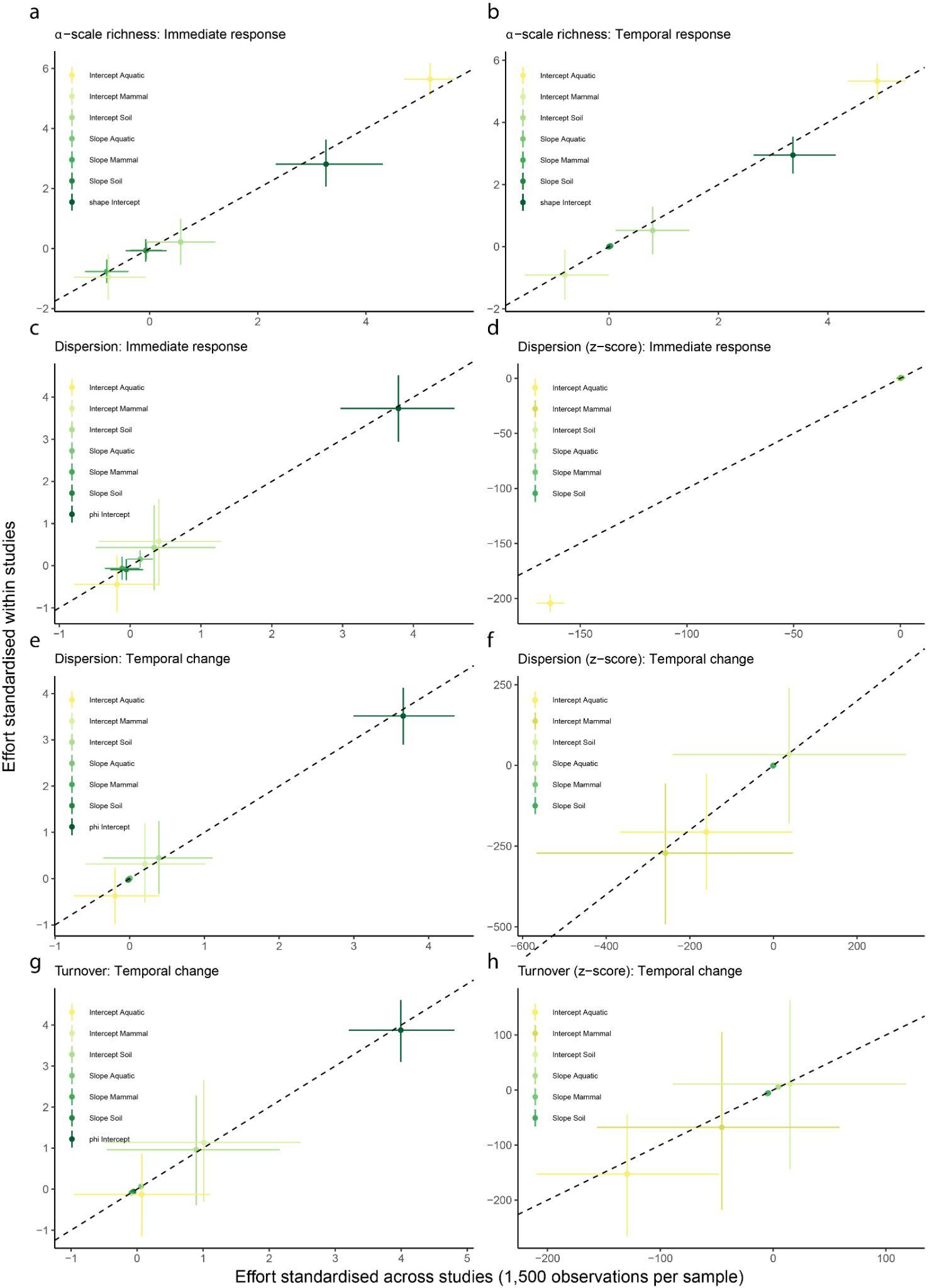


Figure S2: Models fit to data standardized across studies and to data standardized within studies yield very similar parameter estimates. Each panel shows the fixed effect estimates for models fit to (a) richness before-after disturbance, (b) richness change through time following disturbance, (c) dispersion before-after disturbance, (d) dispersion (z-score) before-after disturbance, (e) dispersion change through time following disturbance, (f) dispersion (z-score) change through time following disturbance, (g) turnover change through time following disturbance, and (h) turnover (z-score) change through time following disturbance. Rarefaction performed within time series selected the deepest possible observation depth for each time series or 1500 reads per sample and only mildly increased coverage from 0.96±0.05 to 0.98±0.04. Importantly, sampling depth and sample richness were not correlated.


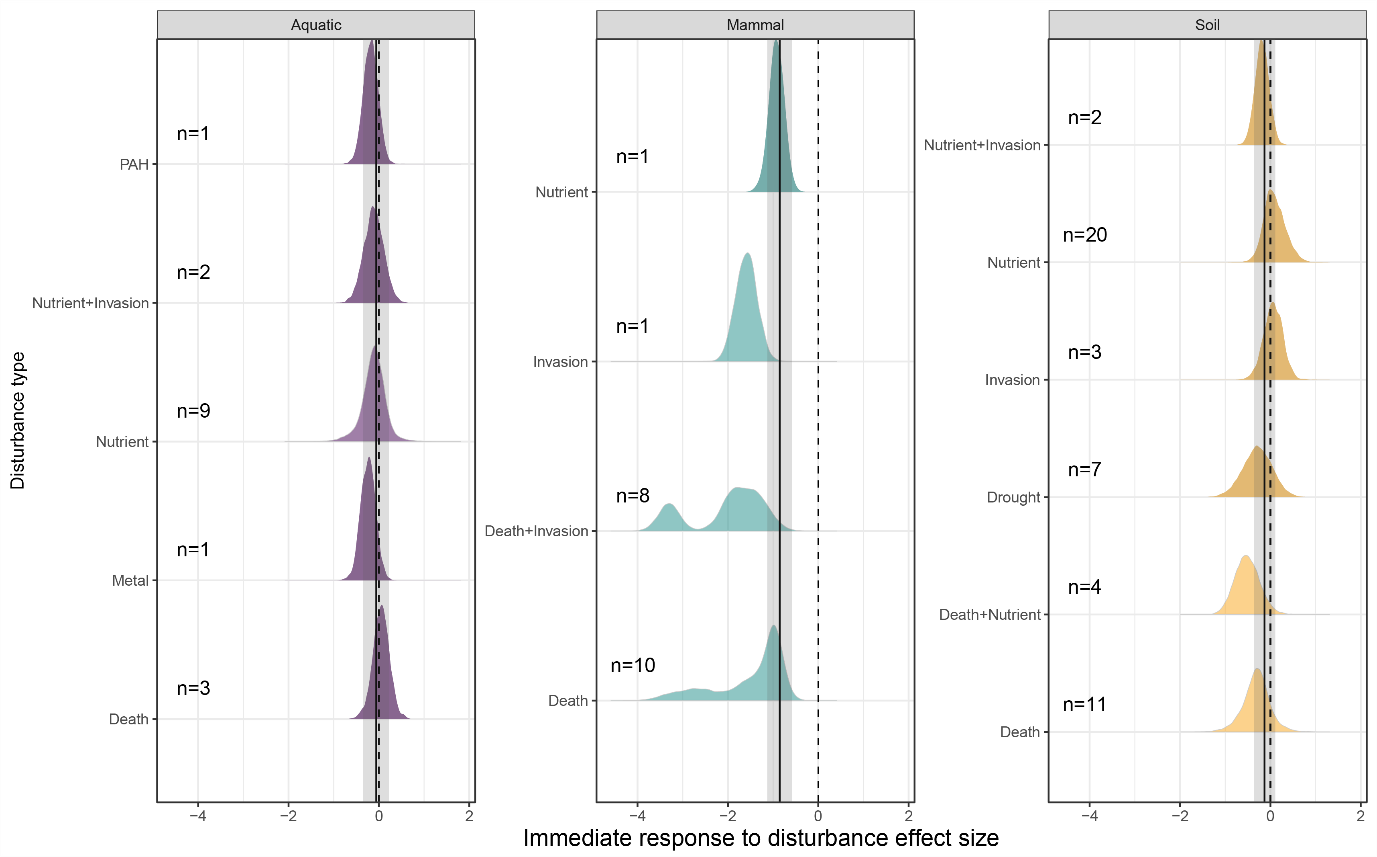
Figure S3. Posterior distributions of the immediate response in richness to disturbance, separated by disturbance type and microbial realm. For each category, n indicates the number of time series included in each category. The dashed line indicates an effect size of 0. Solid lines indicate the mean for the realm, and the shaded area indicates the 95% CI.


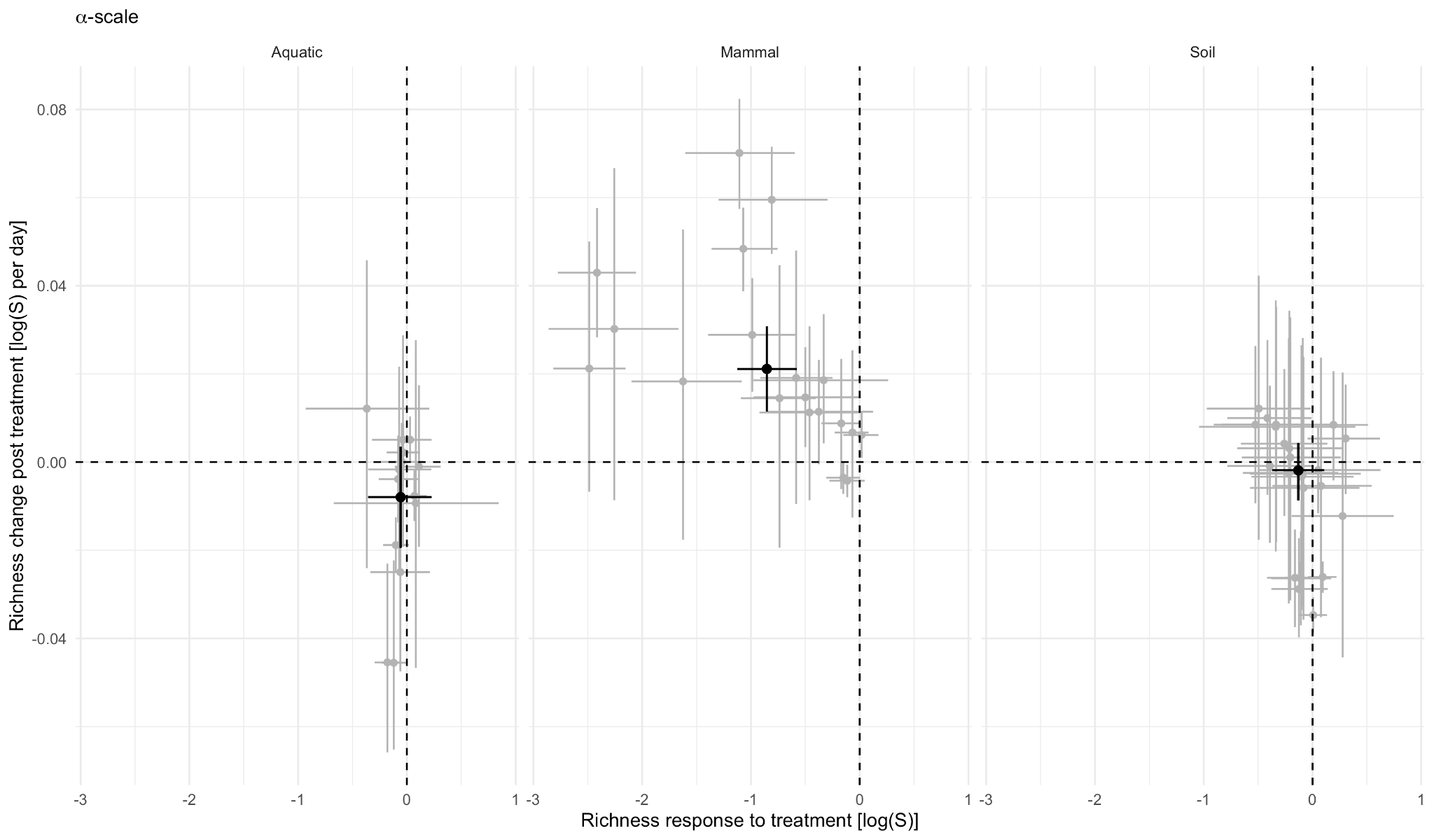


Figure S4. The immediate effect of a disturbance on richness was only related to the rate of recovery of richness in mammals. Each point is a time series, faceted by environment. Immediate richness responses were estimated as the effect of disturbance on log-scale community richness (Figure 3a). Richness response to treatment was calculated as the slope estimates of richness over time. Error bars show the 95% CI for both metrics. Large circles indicate the mean response per environment.


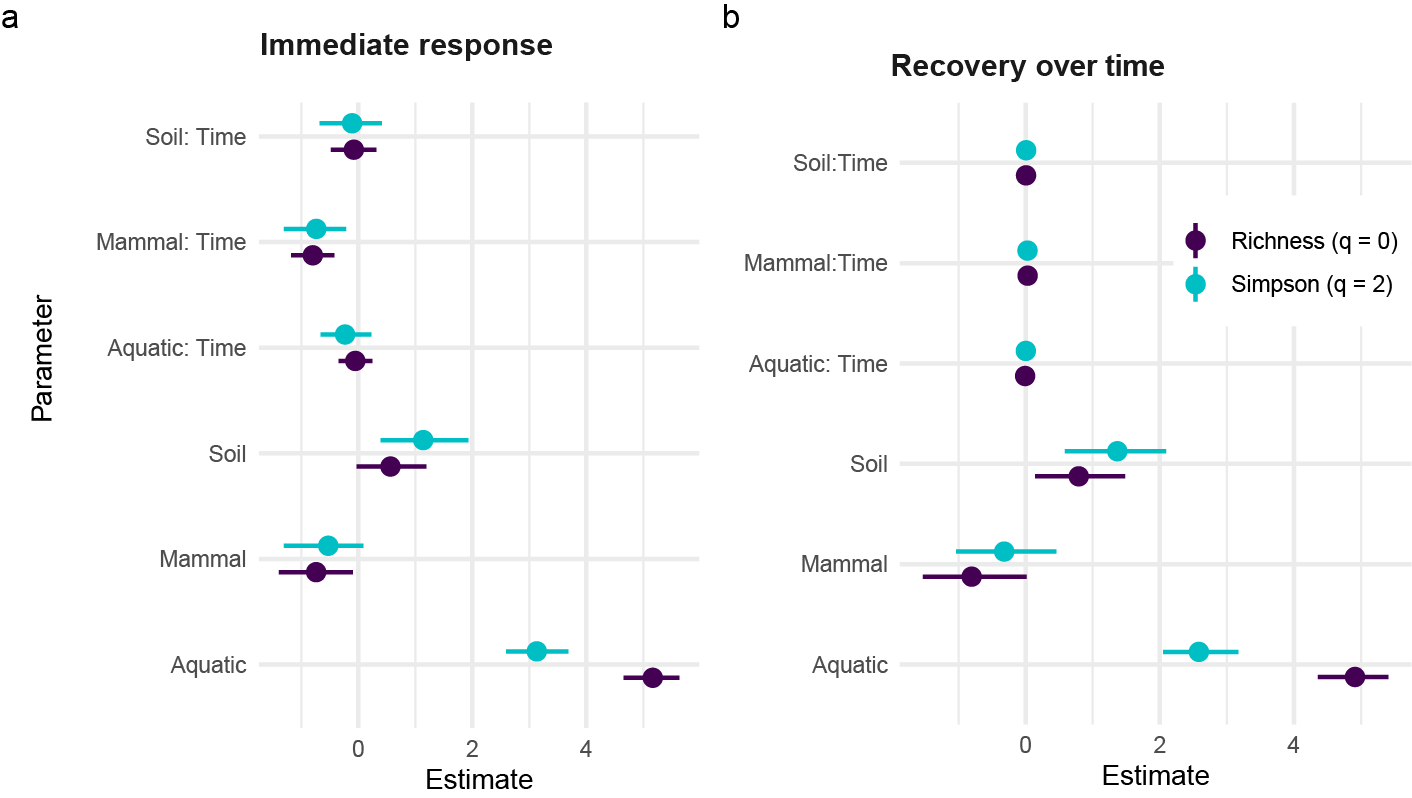


Figure S5. Slope and interval estimate of richness (Hill q_0_, purple) and inverse Simpson’s index (Hill q_2_, blue) immediately following disturbance (a) and over time (b). Error bars represent 95% credible intervals.


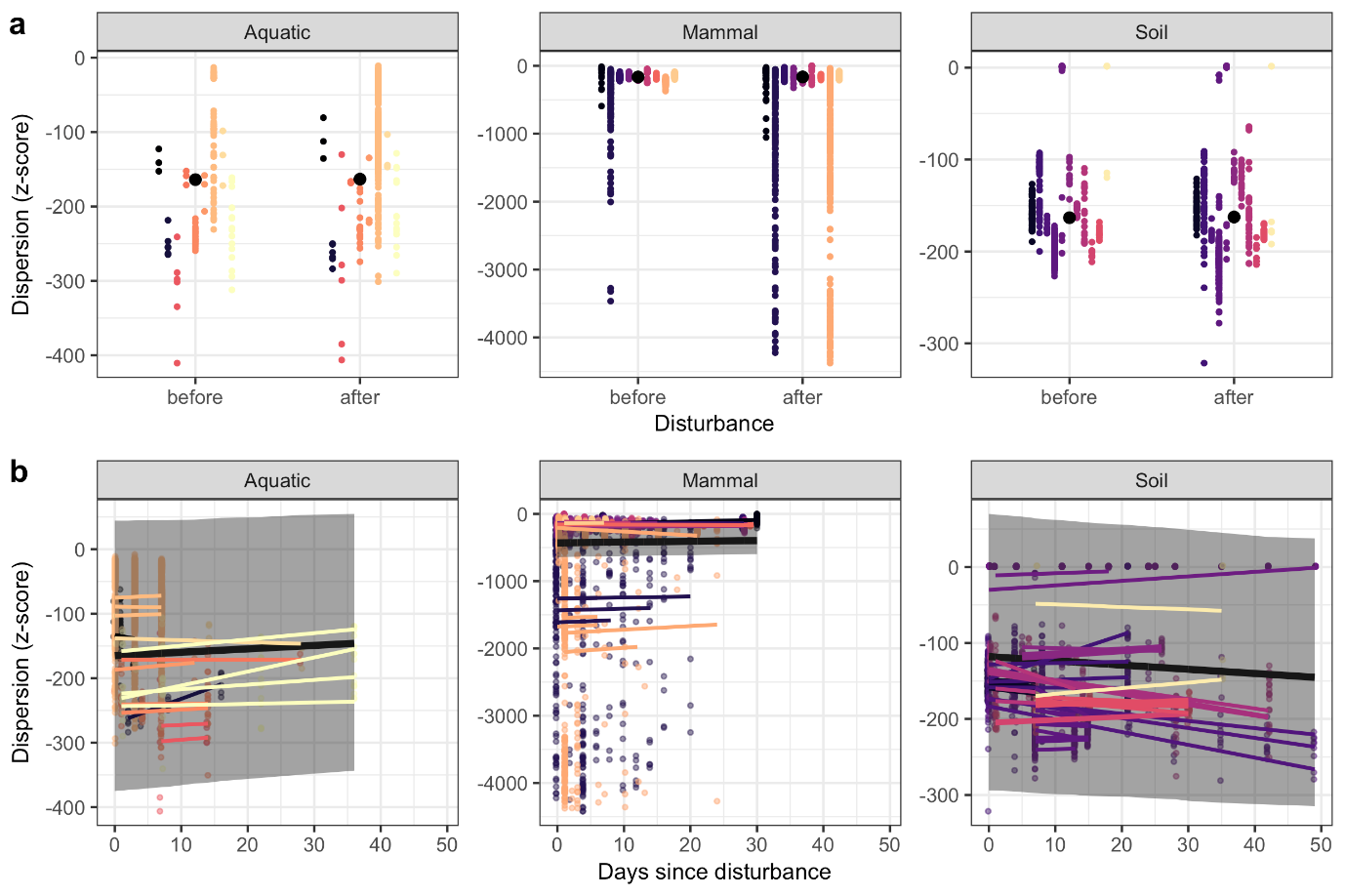


Figure S6. The effect of disturbance on microbiome dispersion, immediately (<4 days) after disturbance (a), and over 50 days of recovery (b). Dispersion was calculated as the pairwise Bray-Curtis distance between replicates for each time point within each time series, and each circle is a Z-score of a pairwise comparison, colored by study. In *a,* solid black circles indicate the mean across time series per environment with a 95% CI indicated by error bars. In *b*, regression lines for each time series are colored by study, and the solid black line shows the mean response across time series per environment. The 95% CI of the overall response in each environment is displayed as a grey shaded area, and environments for which overall trends deviate from zero are indicated with an asterisk (*) on the bottom right corner.


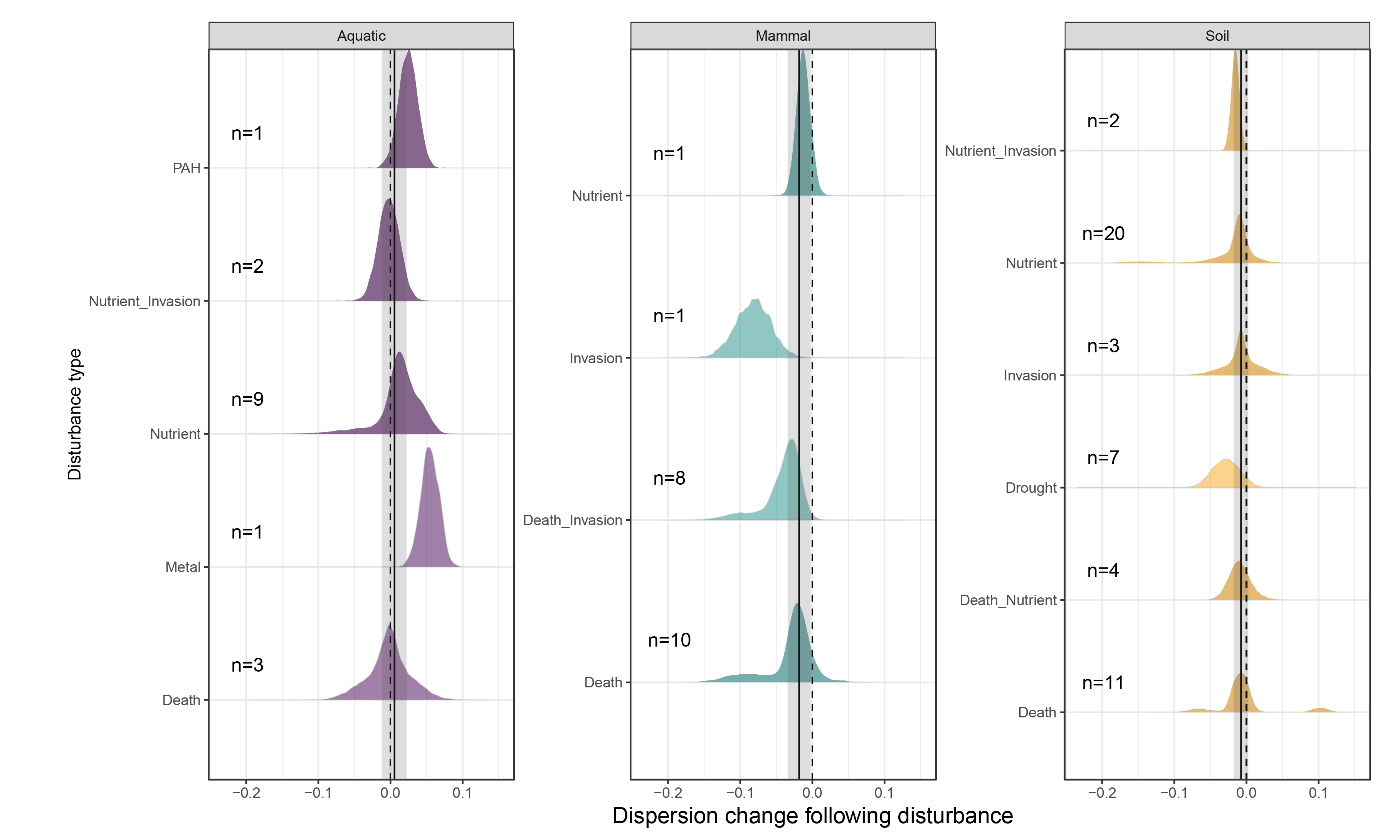
Figure S7. Posterior distribution of temporal response of dispersion to disturbance, separated by disturbance type and microbial realm. For each category, n indicates the number of time series included in each category. The dashed line indicates an effect size of 0. Solid lines indicate the mean for the realm, and the shaded area indicates the 95% CI.


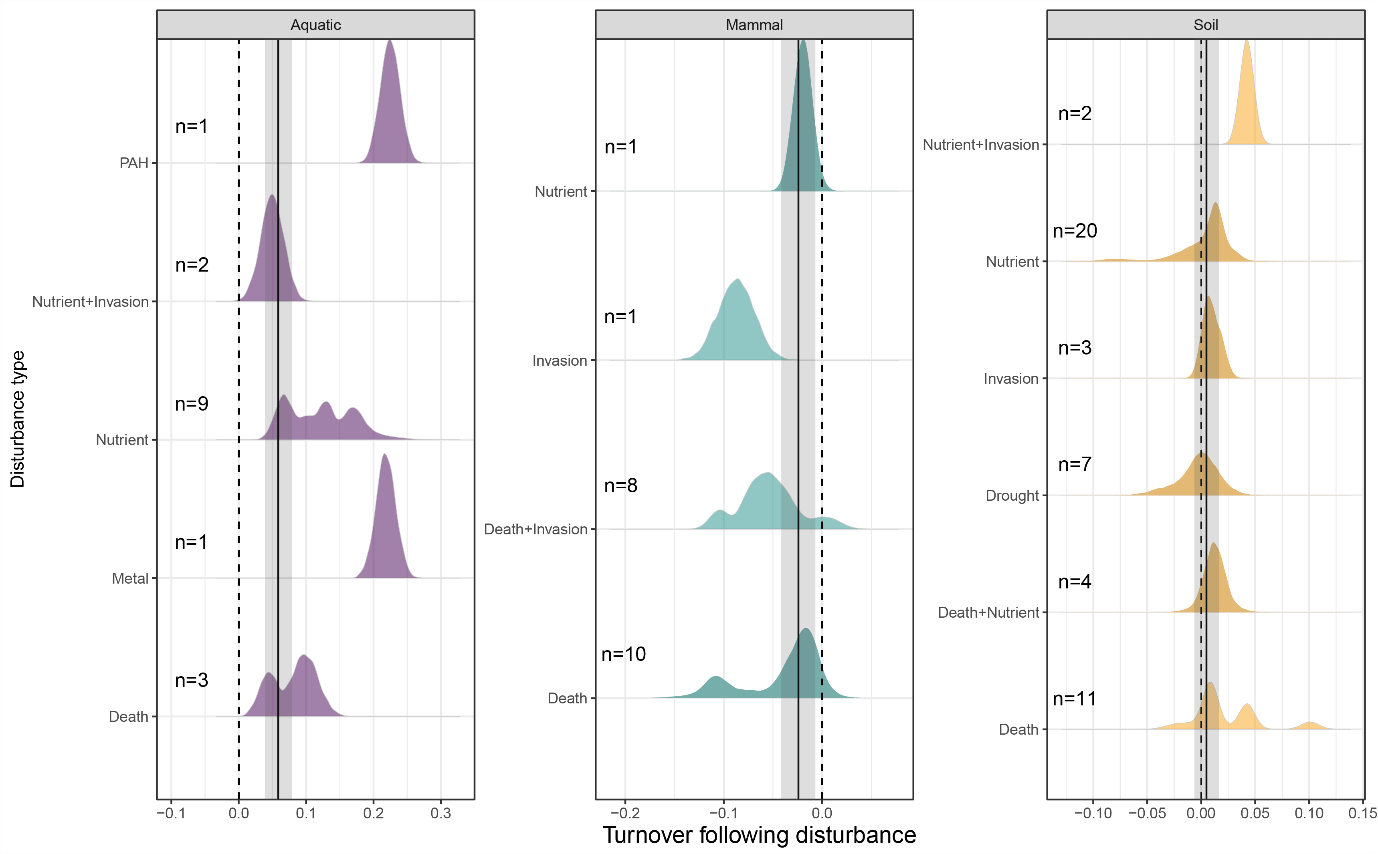
Figure S8. Posterior distribution of temporal response of turnover to disturbance, separated by disturbance type and microbial realm. For each category, n indicates the number of time series included in each category. The dashed line indicates an effect size of 0. Solid lines indicate the mean for the realm, and the shaded area indicates the 95% CI.


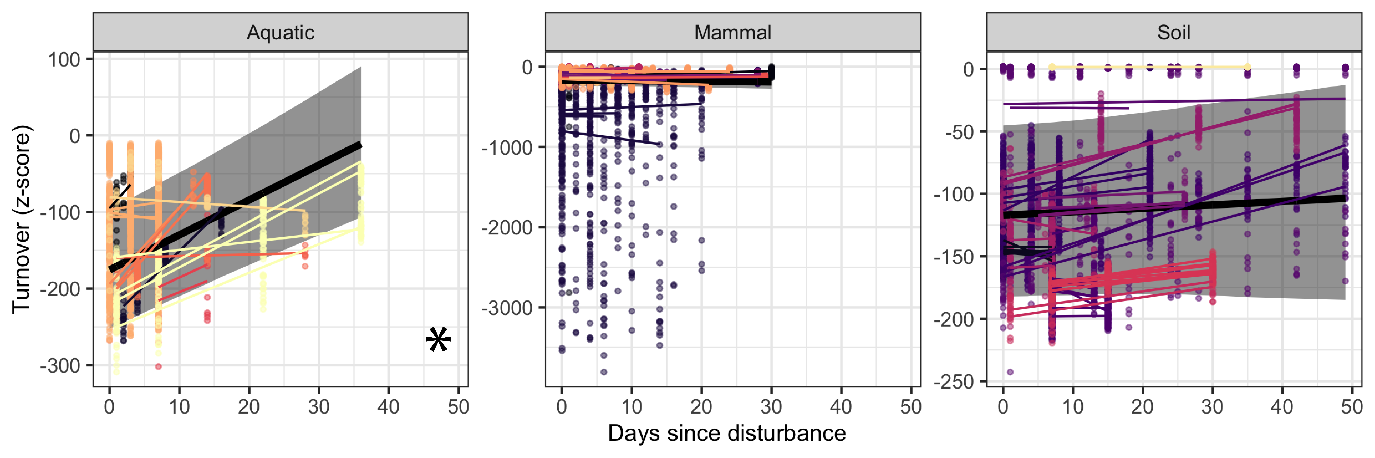


Figure S9. The effect of disturbance on turnover. For each time series, recovery was calculated as the pairwise distance between post-disturbance samples and pre-disturbance controls. Each point is a Z-score of a pairwise comparison, colored by study. Regression lines for each time series are colored by study, and a solid black line indicates the mean response across time series per environment. The 95% CI is displayed as a grey shaded area, and environments for which overall trends deviate from zero are indicated with an asterisk (*) on the bottom right corner.


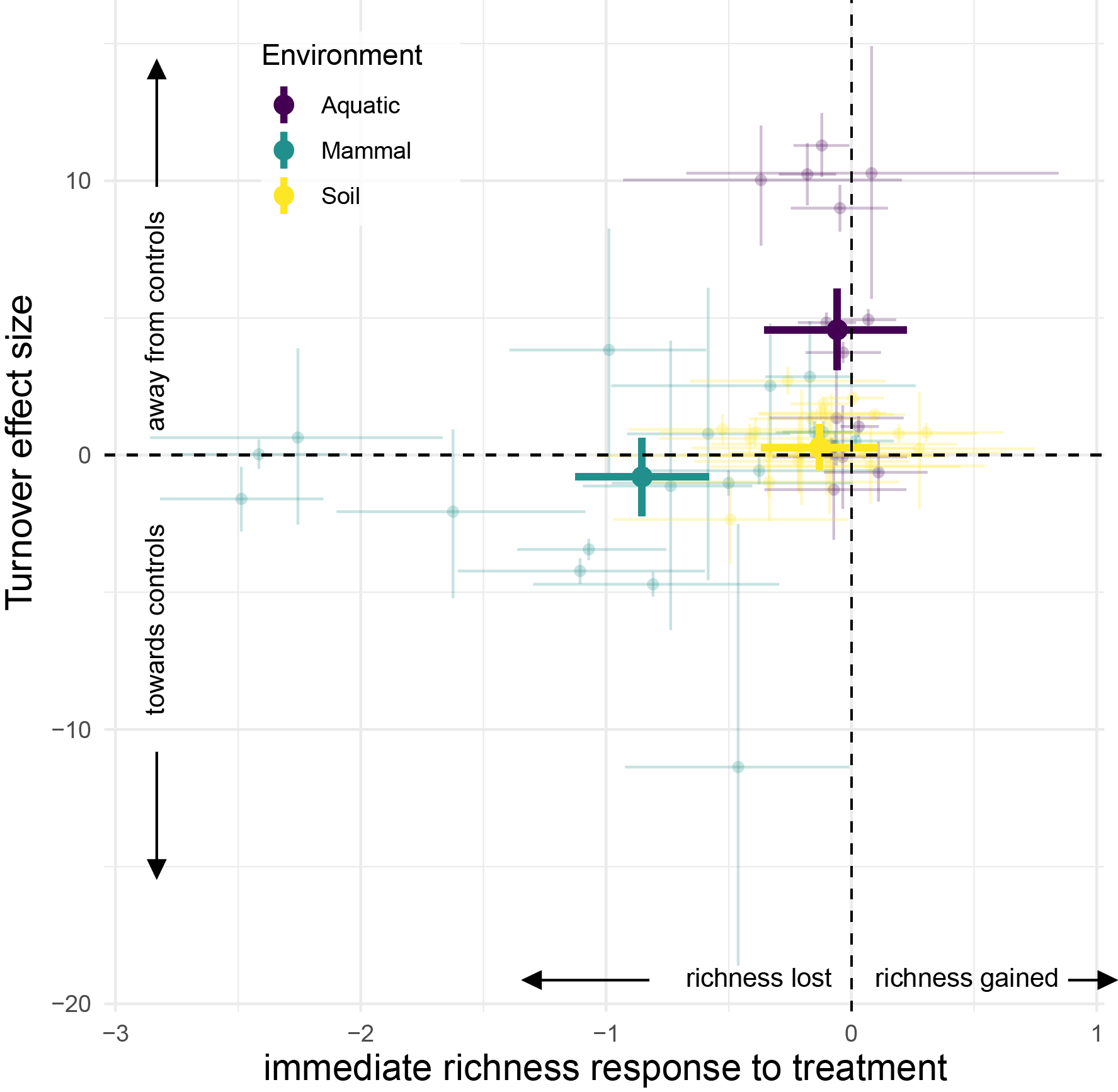


Figure S10.  Relationships between the immediate effect of a disturbance on richness and a microbiome’s long-term recovery of composition vary among environments. Each point is a time series, colored by its environment. Immediate richness responses were calculated as the before-after effect of disturbance on log-transformed community richness (Figure 3a). Turnover rates were calculated as the slope estimates of logit-transformed turnover Z-scores over time. Error bars show the 95% CI for both metrics. Large points indicate the mean responses per environment.
